# Phase Equilibria and Crystallography of Ceramic Oxides

**DOI:** 10.6028/jres.106.059

**Published:** 2001-12-01

**Authors:** W. Wong-Ng, R. S. Roth, T. A. Vanderah, H. F. McMurdie

**Affiliations:** National Institute of Standards and Technology, Gaithersburg, MD 20899-8520

**Keywords:** ceramic oxides, crystal chemistry, crystallography, electronic materials, historical development, phase equilibria

## Abstract

Research in phase equilibria and crystallography has been a tradition in the Ceramics Division at National Bureau of Standards/National Institute of Standatrds and Technology (NBS/NIST) since the early thirties. In the early years, effort was concentrated in areas of Portland cement, ceramic glazes and glasses, instrument bearings, and battery materials. In the past 40 years, a large portion of the work was related to electronic materials, including ferroelectrics, piezoelectrics, ionic conductors, dielectrics, microwave dielectrics, and high-temperature superconductors. As a result of the phase equilibria studies, many new compounds have been discovered. Some of these discoveries have had a significant impact on US industry. Structure determinations of these new phases have often been carried out as a joint effort among NBS/NIST colleagues and also with outside collaborators using both single crystal and neutron and x-ray powder diffraction techniques. All phase equilibria diagrams were included in *Phase Diagrams for Ceramists*, which are collaborative publications between The American Ceramic Society (ACerS) and NBS/NIST. All x-ray powder diffraction patterns have been included in the Powder Diffraction File (PDF). This article gives a brief account of the history of the development of the phase equilibria and crystallographic research on ceramic oxides in the Ceramics Division. Represented systems, particularly electronic materials, are highlighted.

## 1. Introduction

Phase diagrams are critical research tools for many scientific disciplines, including material science, ceramics, geology, physics, metallurgy, chemical engineering, and chemistry. Phase diagrams can be regarded as “road maps” for materials processing. These diagrams contain important information for the development of new materials, control of structure and composition of critical phases, and improvement of properties of technologically important materials. Applications of phase diagrams range from the preparation of high quality single crystals, single-phase bulk materials, deliberate precipitation of second phases, to the formation of melts. Studies of phase equilibria and crystallography of materials are strongly correlated. Crystallographic information is critical for furthering the understanding of phase equilibria, crystal chemistry, physical properties of materials, and performing various theoretical calculations.

Phase equlibria and crystallographic research at NBS/NIST has been an important program since the 1930s. Development of new technologies based on ceramic oxides continued to require new materials and new equipment. As a result, a large number of new phases in a diverse area of material science have been discovered and characterized at NBS/NIST. Methods of structural characterization include single crystal x-ray, powder neutron and x-ray methods, and electron diffraction. Since powder x-ray diffraction patterns are critical for phase identification, patterns of the new phases were also prepared and were included in the Powder Diffraction File (PDF [1]). Throughout these years, NBS/NIST has collaborated with various important ceramic industries, and the systems investigated reflected the changing emphasis of material systems, including cement, glasses, battery materials, dielectrics, ferroelectrics, ionic conductors, superconductors, microwave materials, magnetic materials, and materials for optical applications.

In the following, highlights of the phase equilibria and crystallographic research of oxide materials in the Ceramics Division will be followed by more detailed discussions pertaining to the dielectric and superconductor systems.

## 2. Historical Development

### 2.1 The Early Years (1930s to mid 1960s)

In the twenties, the Clay and Silicate Product Division at NBS (which later became the Ceramics Division) had seven Sections that were all product-oriented. Examples of these sections were “‘Heavy Clay Products”, “White Ware”, “Enamels”, and “Lime and Gypsum”. The functions of these Sections were primarily to develop specifications and to perform testing for government agencies. As an example, bricks were tested for water absorption and for resistance to freezing and thawing as a preliminary to writing specifications. The idea of either “crystallography” or “phase equilibria” of materials was hardly considered. Between the 1930s and mid 1960s, H. F. McMurdie and his colleagues pioneered the studies of the phase relationships and crystallographic aspects of ceramic materials. A portion of the study was also conducted in collaboration with both internal and external laboratories.

#### 2.1.1 Portland Cement

**Fig. 1:**
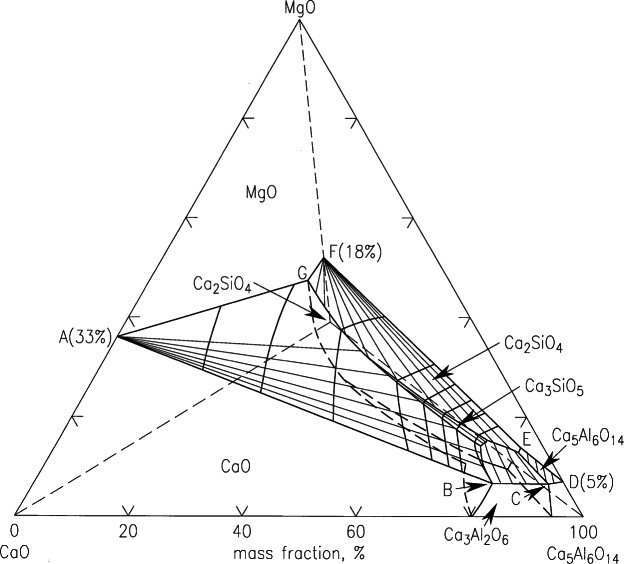
(a) Phase diagram of the system CaO-MgO-2CaO SiO_2_-5CaO 3Al_2_O_3_ [2]; the surface intersecting the sides of the tetrahedron at A-B-C-D-E-F-G indicates the lower level of the primary-phase volume of MgO; C_3_S = 3CaO·SiO_2_; C_2_S = 2CaO·SiO_2_; C_3_A = 3CaO·Al_2_O_3_; C_5_A_3_ = 5CaO·3Al_2_O_3_. 1(b).

One of the early phase diagram studies was reported in 1936 on the formation of MgO in the compositions of Portland cement [2]. Portland cement clinker consists essentially of lime, alumina, silica, ferric oxide, and magnesia. The last two phases may be generally considered as occurring incidentally, as impurities in the basic raw material. Since MgO could be harmful in cement, it would be important to determine its behavior in the cement to establish the composition ranges within which MgO appears as a primary phase (the first crystalline phase to appear on cooling a composition from the liquid state). This work was completed primarily by quenching and petrographic microscopy. Figure 1 shows the phase diagram of the CaO-MgO-2CaO ·SiO_2_-5CaO·3Al_2_O_3_ system. The surface intersecting the sides of the tetrahedron at A-B-C-D-E-F-G indicates the lower level (4 % to 5 %) of the primary phase field (the locus of all compositions in a phase diagram having a common primary phase) of MgO. MgO has an exceptionally large primary phase field.

The structural characterization of the products of hydration of Portland cement was carried out by petrographic and powder x-ray techniques [3] with the phases synthesized by hydrothermal methods. The main finding was that the group of compounds Ca_3_Al_2_Si_3_O_12_ (andradite), Ca_3_Fe_2_Si_3_O_12_ (grossularite), Ca_3_Al_3_H_12_O_12_ and Ca_3_Fe_2_H_12_O_12_ form a complete series of solid solutions, with a garnet structure. The phases with hydrogen were called hydrogarnets. All these compounds were found to be cubic with space group *Ia*3*d*.

#### 2.1.2 Ceramic Glazes and Glasses

In the thirties, information on phases in the PbO-SiO_2_ system was needed in connection with the studies of ceramic glazes and glasses. Four phases were found in this system and their x-ray diffraction patterns were determined by Geller et al. [4], and McMurdie and Bunting [5]. These studies used both x-ray diffraction and petrographic microscopy methods. In addition, x-ray patterns of phases of the K_2_O-PbO-SiO_2_, PbO-SiO_2_, and the Na_2_-PbO-SiO_2_ systems were prepared in cooperation with the Whiteware Section.

**Fig. 2:**
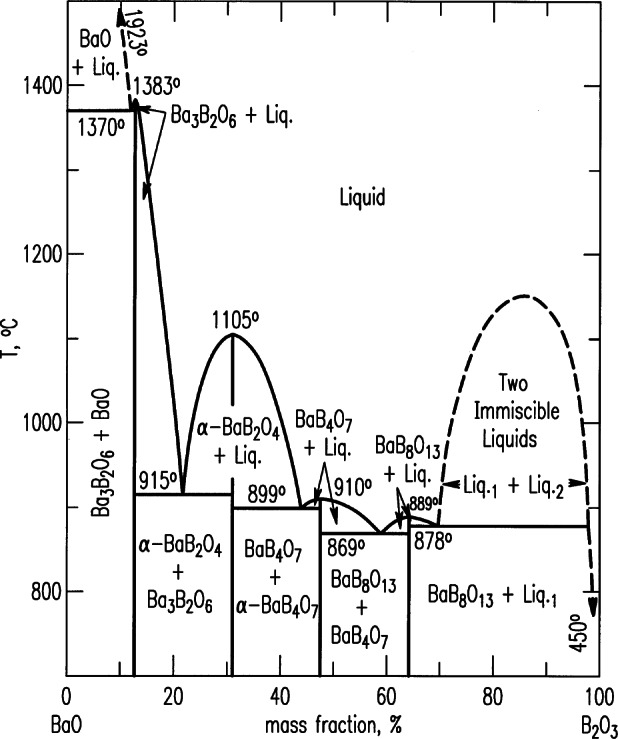
Phase diagram of the system BaO-B2O3 [6]. Liquid immiscibility was observed.

**Fig. 3:**
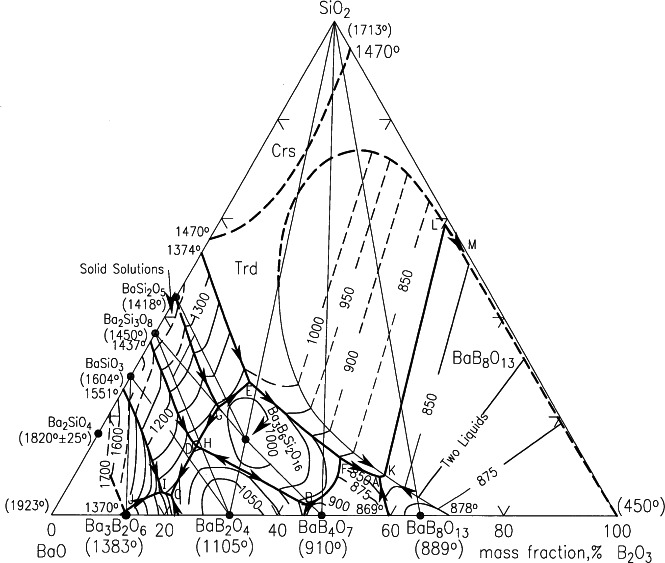
Phase diagram of the system BaO-B_2_O_3_-SiO_2_ [7].

**Fig. 4:**
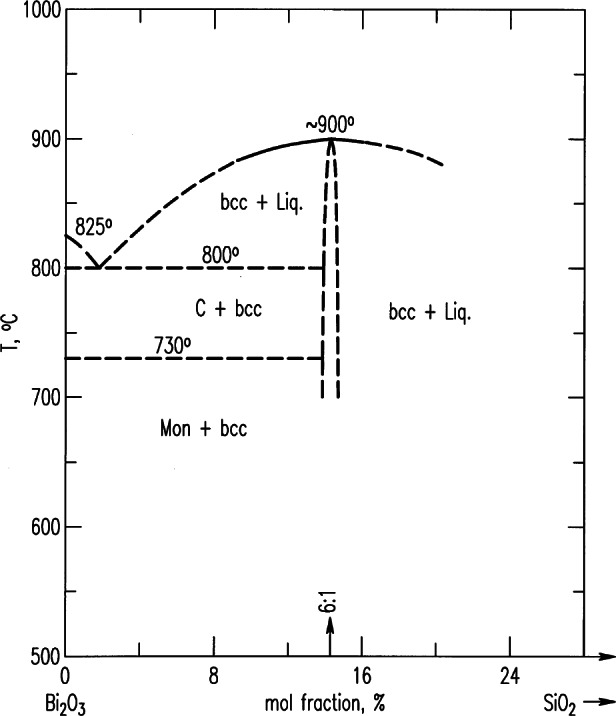
Phase diagram of the system Bi_2_O_3_-SiO_2_ [34].

In the late forties, phase equilibrium information for the system BaO-B_2_O_3_-SiO_3_ and the subsystem BaO-B_2_O_3_ [6] were of importance to the glass industry as a starting point for the investigations of barium crown glasses (see Fig. 2). These glasses are characterized by high refractive index and low dispersion. The experiments included quenchings and the use of differential thermal analysis (DTA). Phases were identified by microscopy and x-ray diffraction. X-ray patterns of four phases including the low form of BaB_2_O_4_ were reported in the BaO-B_2_O_3_ system. A two-liquid region (liquid immiscibility) was found. In the ternary system BaO-B_2_O_3_-SiO_2_ system [7], one ternary phase, Ba_3_B_6_Si_2_O_16_, was discovered and a large two-liquid region was also located (Fig. 4). The system BaO-SiO_2_ was later modified by Roth and Levin [8] to show the existence of several new compounds between BaO·2SiO_2_ and 2BaO·3SiO_2_ in the region labeled as “solid solutions” in Fig. 3.

In the fifties, the condition of immiscibility in borate and silicate systems and its frequent occurrence had been noted by J. W. Greig and F. C. Kracek at the Geophysical Laboratory. Since it was important as a commercial and scientific problem to understand the basic causes of liquid immiscibility, Levin and Block [9, 10] attempted to interpret this phenomenon quantitatively by applying crystal-chemical principles to nineteen binary and eight ternary glass systems. Using ionic radii and one of the two coordination types (for glass-former cations and for modifier cations), they found that the additive density method gives agreement with experimental results in both the borate and silicate systems to within a mole fraction of 5 %. The oxygen-volume method agrees to within 2 % (mole fraction).

In the early sixties, in order to assist the U.S. industry to develop new compositions and search for better property for optical glass, the system La_2_O_3_-B_2_O_3_ was studied by Levin et al. [11] by the quenching method. Phases were identified by petrographic microscopy and x-ray diffraction. Three binary compounds were found and no solid solution was encountered in this system. LaBO_3_ was of aragonite type with a polymorphic change at 1488 °C to a form similar to calcite. A two-liquid region was found but no solid solution was detected.

#### 2.1.3 Properties of Materials for Jewel Instrument Bearings

Around the mid-forties, in the course of an investigation on jewel bearings for instruments, conducted under authorization of the Bureau of Aeronautics, United States Navy, NBS made a number of tests and examinations of various materials, included corundum (natural and synthetic), synthetic spinel and glass [12]. The natural corundum samples originally were from Montana. Synthetic corundum was prepared by the Verneuil Method. The properties measured were homogeneity, structural defects, hardness, and strength. It was found that although the corundum has the greatest hardness and strength, its hardness is much greater than that of the usual steel instrument pivots which would cause the pivots to deform or rust. Glass has about the same hardness as the pivots but glass bearings may break under vibration and impact. Spinel is intermediate between corundum and glass in strength and hardness.

#### 2.1.4 Battery Materials

In the mid-forties, because of World War II, dry cells were put to many new uses involving conditions of extreme heat and cold, and storage over long periods of time. At the request of the Electrochemical Section a study was conducted to characterize a large number of dry battery materials (mainly MnO_2_), related synthetic materials and natural minerals [13]. This task showed an early example of combining a number of crystallographic methods, namely, x-ray diffraction (both at room temperature and at elevated temperatures [14] for phase identification and phase transformation studies), DTA for the study of thermal events, and the electron microscope for particle size and surface characterization. MnO_2_ was found to lose oxygen at about 600 °C and 950 °C to bixbyite (Mn_2_O_3_) and Mn_3_O_4_ (hausemannite). A reversible polymorphic change from the tetragonal hausemannite to a spinal form was also determined.

Related to the work on battery materials, a characterization study was conducted on the phases formed during the discharge of cells. The main Mn-containing phase found after discharge was hetaerolite (ZnMn_2_O_4_) in which the valence of Mn is 3. A smaller amount of ZnCl_2·_4Zn(OH)_2_ was also found [15,16].

#### 2.1.5 Uranium Dioxide with Metal Oxides

In the mid-fifties, investigation of the high-temperature reactions of uranium with a large variety of materials comprised an important segment of the research activities of the U.S. Atomic Energy Commission and many of its contractors. In the Porcelain and Pottery Section of the NBS, a project for the determination of the phase-equilibrium relations of binary systems containing UO_2_ and various metal oxides was carried out by Roth et al. [17]. This study also included a critical review of the phase relations at high temperature of UO_2_ with 15 other oxides (BeO, MgO, CaO, SrO, BaO, CuO, Al_2_O_3_, Y_2_O_3_, Nd_2_O_3_, SiO_2_, ZrO_2_, SnO_2_, CeO_2_, ThO_2_, V_2_O_5_). As part of this work, the technology was developed for ZrO_2_ clad VO_2_ fuel pellets, used to this day for the fuel rods in all modern nuclear reactors.

### 2.2 Period of 1960s to late 1980s

After the retirement of H. F. McMurdie in 1966, the Crystallographic Section of the Ceramics Division was headed by S. Block, and the Phase Equilibria Section was headed by R. S. Roth. The program of the crystallographic section was divided into several long-term disciplines [18]: 1) structure determinations (e.g., a great number of borates and inorganic complexes [19,20]), 2) high pressure (see High Pressure Crystallography), 3) powder diffraction (primarily the determination of standard patterns with the JCPDS-ICDD (see JCPDS-ICDD collaborative program), and 4) the Crystal Data Project (supported by the Standard Reference Data Program) [21].

During this period, R. S. Roth and his co-workers made significant contributions to the phase equilibria and crystal chemistry research of a great variety of important classes of ceramics (over 200 publications today). Of special note was the paper on the classification of ABO_3_ compounds with emphasis on the perovskite structure type phases [22]. In order to understand the crystal chemistry of these phases in detail, many single crystals were grown. As crystal growth of the high-temperature oxides was not a straight-forward task due to the high melting temperature and the incongruent melting nature of most oxides in the systems of interest, a low-temperature flux was often used as an aid. Crystallographic investigations were largely carried out involving international collaborations (A. D. Wadsley, S. Anderson, N. C. Stevenson, B. M. Gatehause, I. E. Grey, etc.). Selective studies of the phase equilibria, crystal chemistry and crystallography of important ceramics during this period are summarized below.

#### 2.2.1 Ferroelectric Materials

The viable applications of BaTiO_3_ in various fields of industry, including the ferroelectric with the simplest structure, has made this compound a subject of much investigation for many years. Roth et al. conducted a detailed study of the phase diagram of the BaTiO_3_-TiO_2_ system, which will be discussed in the section of the dielectric materials [23]. Other Ba-containing materials being studied that are of potential ferroelectric applications systems included Ba_6_Nb_28/3_Ni_2/3_O_30_ [24] and (Ba_6−2_*_x_*R_2_*_x_*)(Nb_9−_*_x_*Fe_1+_*_x_*)O_30_ [25].

Because of the ferroelectric properties of the compound Cd_2_Nb_2_O_7_ below room temperature, the phase equilibria of the CdO-Nb_2_O_5_ system was of interest [26]. Based on the pyrochore type structure of Cd_2_Nb_2_O_7_, Roth has also surveyed the reactions occurring in binary oxide mixtures of the type A_2_O_3_:2BO_2_ as part of the program on ferroelectric ceramics (A = La, Nd, Sm, Gd, Bi, Y, Dy, Yb, In, Sb; B = Ce, U, Ti, Sn, and Zr) [27]. On the basis of the existence of the two compounds, La_2_O_3_·2ZrO_2_ and Nd_2_O_3_·2ZrO_2_, the phase diagrams for the systems La_2_O_3_ – ZrO_2_ and Nd_2_O_3_ – ZrO_2_ were also determined.

Another system of interest was PbO-Nb_2_O_5_ [28] because of the orthorhombic ferroelectric modification phase of PbNb_2_O_6_ [29]; there were contradictory literature reports about the phase transformation of this phase. High-temperature x-ray patterns indicated that pure PbO·Nb_2_O_5_ has a tetragonal symmetry as a stable modification from the temperature of the high-low phase transformation to the melting point. If the high-temperature modification is cooled quickly from below the transformation point, it will maintain the tetragonal structure in a metastable condition. When it reaches the Curie point of 590 °C [30], it transforms metastably and reversibly to the orthorhombic ferroelectric modification.

#### 2.2.2 Piezoelectric Ceramics

Piezoelectric properties of BaTiO_3_ ceramic have simulated a search for other ferroelectrics suitable for fabricating piezoelectric ceramics. Of particular interest was to discover materials having electrical mechanical properties that are stable through a wide range of temperature. Roth’s work in the area of piezoelectric ceramics was extensive. In the mid-fifties, Roth et al. studied the systems PbZrO_3_-PbTiO_3_, PbTiO_3_-PbO:SnO_2_, PbTiO_3_-PbZrO_3_-PbO:SnO_2_, and PbTiO_3_-PbHfO_3_ [31,32], and discovered the possible desirable properties of compositions near a morphotropic transformation between ferroelectric solid-solution phases. Piezoelectric properties of the lead zirconate-lead titanate solid solution that was discovered in the PbZrO_3_-PbTiO_3_ system [32], now known as PZT, have made PZT one of the most important advanced electronic ceramic materials known to this day. This material also revolutionized various industries with its diverse applications.

Three series of partial phase diagrams of the Bi_2_O_3_-MO*_x_* systems (*x* = 1, M = Ni, Zn, Cd, Mg, Ca, Sr, Ba, Pb; *x* = 1.5, M = B, Al, Ga, Fe, Mn, Sb, Lu, Sm, La; *x* = 2, M = Si, Ge, Ti, Sn, Zr, Ce, Te) were determined in order to determine the influence of various foreign cations on the polymorphism of Bi_2_O_3_ [33,34]. As these phases were reported to melt congruently they became ideal candidates for crystal growth. Fig. 4 gives an example of the diagram of the Bi_2_O_3_-SiO_2_ system. This study resulted in the development of a sought-after piezoelectric material, SiBi_12_O_20_, a phase with the largest known rotary inversion of any oxide compound at the time, and is of considerable commercial interest. A structural analysis proved that the phase was noncentrosymmetric. Small tetravalent ions were found to stabilize the cubic body-centered phase. Body centered cubic bismuth oxide is now believed to be a mixed valence compound with a formula Bi^+5^_0.5_Bi^+3^_12.5_O_20_. Roth et al. also found that BaO, SrO, and CaO entered into the solid solution causing an increase in the melting point of Bi_2_O_3_. This solid solution with apparent rhombohedral symmetry was found to have interesting oxygen ion conductivity.

#### 2.2.3 Solid State Ionic Conductors

In the eighties, there was a significant interest in materials which undergo topotactic insertion of lithium because of their potential use as electrode materials in secondary batteries [35,36]. Lithium is ionic in these compounds, and the charge is compensated by a reduction of the host cations. The host structures may be of the layer or framework type. In general, in the framework type structure, Li^+^ ions occupy formerly vacant cation sites, and in the layer type structure, they are accommodated in the van der Waals gap between layers.

In collaboration with the Bell Telephone Laboratories, two structure types and their derivatives were found to be particularly suitable for lithium insertion reactions, namely, rutile structure (TiO_2_) related oxides, and ReO_3_ type structure [35,36]. The crystal chemistry and structure of a large number of lithium-inserted metal oxides have been studied [32–52]: Li*_x_*ReO_3_ (*x* = 1,2), Li_0.35_ReO_3_, Li_2_FeV_3_O_8_, Li_2_SnO_3_, Li_2_ZrO_3_, Li_3_TaO_4_, LiTa_3_O_8_, Li_2_FeV_3_O_8_, Li_0.5_TiO_2_, LiTi_2_O_4_, Li_·325_La_·5625_MoO_4_, δLiV_2_O_5_, Li_6_Zr_2_O_7_, Ba_8_(Me_6_Li_2_)O_24_ (Me = Nb, Ta), Ba_10_(W_6_Li_4_)O_20_, and K*_x_*Li*_x_*Ti_4−2/_*_x_*O_8_. The essential features of the structures are chains of occupied or vacant face-shared MO_6_ octahedra stacking along the hexagonal *c*-axis. Neighboring stacks of face shared octahedra only share corners. It was found that in compounds with Li contents equal to or greater than one in Li-inserted ReO_3_, the ReO_3_ host lattice undergoes a twist about the shared corners of the ReO_6_ octahedra to accommodate the coordination preferred by the Li ions.

Another area of study was the search for suitable window materials for high-power infrared lasers. There was also a need for non-centrosymmetric crystals with large birefringence which may exhibit second harmonic generation and allow optical mixing in the infrared. Studies on heavy metal halides showed that mercurous chloride single crystals could be grown of a size and perfection suitable for prism polarizers with transmission to at least 16 μm [53]. In addition, a study of the TlCl-PbCl_2_ system was conducted and it was found that the compound 3TlCl·PbCl_2_ ((Tl_0.75_Pb_0.25_)_4_Cl_5_) which has a cation-disordered structure can enhance chloride ion conductivity by doping with TlCl.

## 3. Phase Equilibria and Crystallographic Studies of Dielectric/Microwave Materials

### 3.1 1950s to Early 1990

Roth and his coworkers started the investigation of the phase equilibria of dielectric materials in the 1950s. The main goal of these studies was to search for new materials with high dielectric constants and to understand the phase relationships and structural nature of these ceramic oxides. The required properties for ceramic microwave materials include high dielectric constant or permittivity, minimal dielectric loss (high quality factor, *Q*), and essentially zero temperature coefficient (*T*_f_). These studies were focused on the tantalate, niobate, titanate and tungstate systems, and their chemistry with alkaline-earth and rare-earth oxides. The structure of most of these niobates, tantalates, and titanates consists of octahedral coordination that would likely produce high dielectric constants. Selected systems being studied from the fifties to the early 1990s include BaO-TiO_2_ [23], BaO-Nb_2_O_5_ [54], Nb_2_O_5_-WO_3_ [55], Ta_2_O_5_-TiO_2_ [56], Ta_2_O_5_-WO_3_ [57], Ta_2_O_5_-ZrO_2_ [57], ZrO_2_-Nb_2_O_5_ [58], Ta_2_O_5_-WO_3_-Al_2_O_3_ [59], ZrO_2_-TiO_2_ [60], BaO-CoO-O-CO_2_ [61], BaO-ZnO-TiO_2_ [62], BaO-TiO_2_-Nb_2_O_5_ [63,64], and BaO-Nd_2_O_3_-TiO_2_ [65]. Of special importance was the discovery and structural analyses of the multiple phase formation (homologous series) of Nb_2_O_5_ with either TiO_2_ or WO_3_ [55,66] as opposed to the solid solution of these aliovalent ions previously proposed. This may be compared to the structural analysis of the incommensurate solid solution described for the systems of Ta_2_O_5_ with TiO_2_ and WO_3_ [56,57], as well as ZrO_2_ with Nb_2_O_5_ or Ta_2_O_5_ [58].

#### 3.1.1 BaO-TiO_2_

**Fig. 5:**
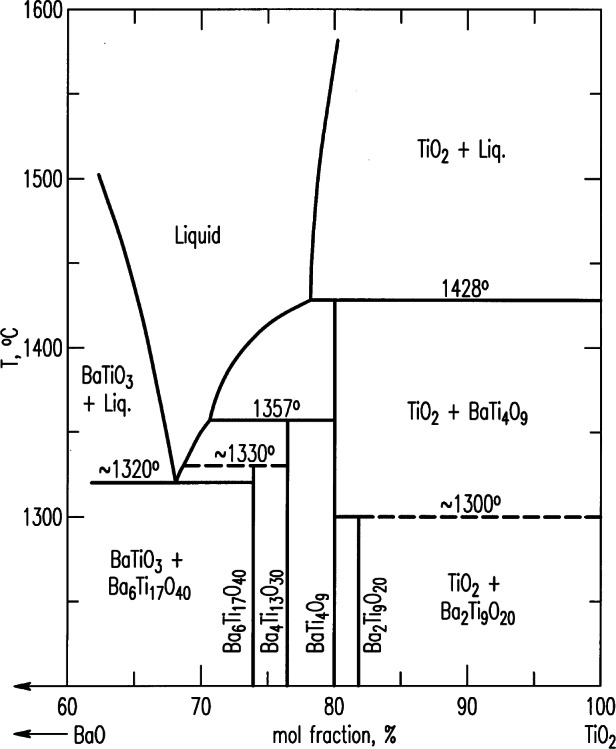
Phase relations in the BaO-TiO_2_ system for compositions with > 60 % mole fraciton TiO_2_ [23]. Abbreviations and symbols used include: BT (BaTiO_3_); B_6_T_17_ (Ba_6_Ti_17_O_40_); B_4_T_13_ (Ba_4_Ti_13_O_30_); BT_4_ (BaTi_4_O_9_); B_2_T_9_ (Ba_2_Ti_9_O_20_); L (liquid); (•) solid phases, quenched; (o) melted, quenched.

The phase diagram of the BaO-TiO_2_ system determined by R. S. Roth et al. and his colleagues in 1974 [23] has provided key data for understanding and processing barium titanate dielectric ceramics important for wireless communications technology. Figure 5 shows the phase relationships in the BaO-TiO_2_ system for compositions with > 60 % mole fraction TiO_2_. As a result of this work, four stable phases (Ba_6_Ti_17_O_40_, Ba_4_Ti_13_O_30_, BaTi_4_O_9_, and Ba_2_Ti_9_O_20_) and two low temperature phases (BaTi_2_O_5_ and BaTi_5_O_11_) were discovered. Commercial devices in the microwave industry often use the BaTi_4_O_9_ and Ba_2_Ti_9_O_20_ polytitanates as the major constituents in high frequency dielectric ceramics (i.e., microwave-filter devices). A full structural investigation was given for Ba_4_Ti_13_O_30_, and defect intergrowth of Ba_2_Ti_9_O_20_ [67] and BaTi_5_O_11_ [68] were characterized in detail.

#### 3.1.2 BaO-TiO_2_-MO*_x_* (M = metals)

Most of microwave materials used nowadays are titanate based ceramics. Various ternary systems involving BaO and TiO_2_ have been studied due to the technical importance of both ferroelectric BaTiO_3_ and the microwave dielectric properties of the barium polytitanates, BaTi_4_O_9_ and Ba_2_Ti_9_O_20_. The ferroelectric properties of the ABX_3_ perovskites can be modified by incorporation of other ions in solid solution and vacancy distribution on selected atomic sites of the structure. The dielectric properties of phases are additive so that the inclusion of one or more other phases in equilirium with the polytitanates can be used to modify the electronic properties of dielectric ceramics. Practically all ceramic components are processed as mixtures, that is, with controlled amounts of second phases to achieve a net-overall zero temperature coefficient. Because of the technological importance of these systems, a review of the phase equilibria and crystal chemistry of the binary and ternary barium polytitanates involving the addition of mostly aliovalent ions to barium polytitanates has been reported by Roth et al. [62]. In this review, an extensive discussion of the fundamental crystal chemistry and crystal structures of various polytitantes have also been given. The various binary and ternary systems discussed included BaO-ZnO, ZnO-TiO_2_, BaO-TiO_2_, BaO-TiO_2_-ZrO_2_, BaO-TiO_2_-Al_2_O_3_, BaO-TiO_2_-MgO, BaO-TiO_2_-Nb_2_O_5_, BaO-TiO_2_-ZnO. Among these systems, two are highlighted in the following.

##### 3.1.2.1 BaO-TiO_2_-ZnO System

**Fig. 6:**
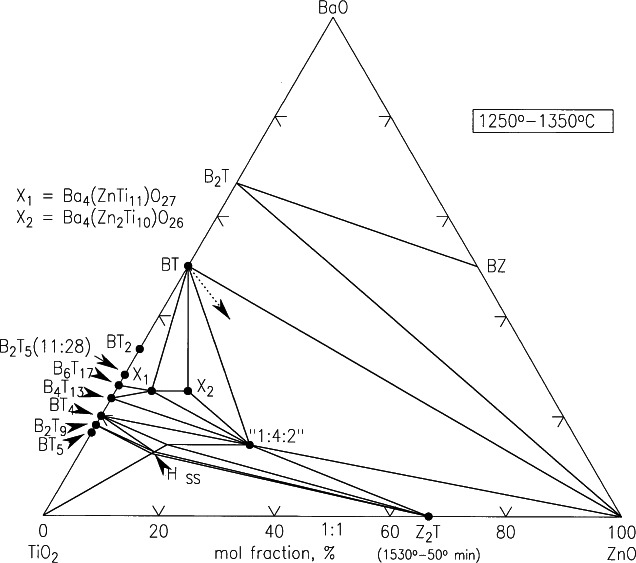
Phase Equilibria diagram of the system BaO-TiO_2_-ZnO [62]. Filled circles represent compounds stable at 1250 °C. Open circles represent compositions of most of the specimens prepared for the study. Dotted arrow represents the apparent direction of the maximum amount of solid solution of ZnO in BaTO_3_.

The phase diagram of the BaO-TiO_2_-ZnO system was of interest (Fig. 6) because it was reported that the composition Ba_3_Ti_12_Zn_7_O_34_ has a positive temperature coefficient of the dielectric constant, and therefore can be used to compensate the negative coefficient in ceramic specimens of BaTi_4_O_9_ [62]. No solid solution of ZnO in any of the binary barium polytitanates was observed. The system contains at least four ternary phases: Ba_x_Zn_x_Ti_8−x_O_16_ with the Hollandite structure, Ba_4_ZnTi_11_O_27_, Ba_2_ZnTi_5_O_13_, and BaZn_2_Ti_4_O_11_. The structure of Ba_4_ZnTi_11_O_27_ and Ba_2_ZnTi_5_O_13_ were investigated at NIST by the single crystal x-ray technique.

**Fig. 7:**
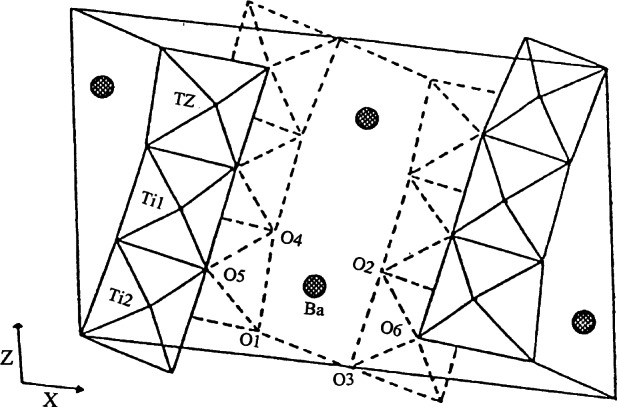
Structure of Ba_2_ZnTi_5_O_13_ [62]. TZ is used to indicate the mixed (Ti, Zn) site.

The projection of the structure of Ba_2_ZnTi_5_O_13_ along the *b*-axis is shown in Fig. 7 (Monoclinic *C*2/*m*, *a* = 15.236(2) Å, *b* = 3.8992(7) Å, *c* = 9.139(2) Å, *β*= 98.78(2) Å). This compound, which was found to be isostructural with K_2_Ti_6_O_13_, crystallizes in the form of thin sheets. The short *b*-axis corresponds to the axial O-Ti-O distance in a TiO_6_ octahedron. The opened structure can be described as consisting of zigzag ribbons of (Ti,Zn)O_6_ octahedra running along *c*. Each repeating unit in these ribbons comprises of a set of three edge-sharing octahedra connected to three other edge-sharing octahedra at a level of one-half unit cell down (and up) along *b*. Rectangular open channels of approximate size 3.2 Å by 2.9 Å can be seen running parallel to *b*. Zn was found to substitute partially for Ti in octahedral sites in this structure.

**Fig. 8:**
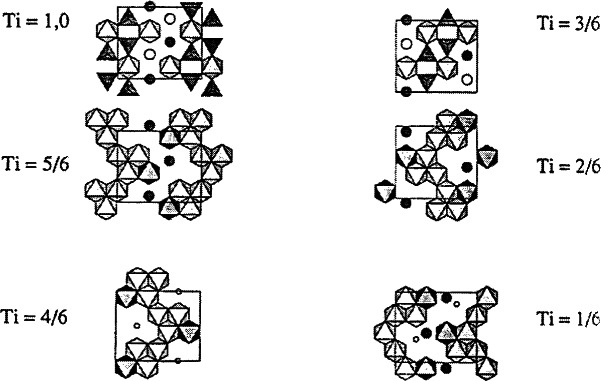
Ideal representation of the crystal structure of BaZn_2_Ti_4_O_11_ at the six (Ti,Zn) levels of the unit cell [62]. Dark triangles represent ZnO_4_ polyhedra pointing up ad down and dark octahedra represent the “ZnO_6_” octahedra.

The overall structure of BaZn_2_Ti_4_O_11_ (Orthorhombic *Pbcn*, *a* = 14.140(3) Å, *b* = 11.592 (2) Å, and *c* = 11.1173 (13) Å) consists of a three-dimensional network of distorted, edge-sharing and corner-sharing octahedra with Zn filling some tetrahedral interstices. Ti atoms were found to occupy only octahedral positions. Similar to Ba_2_ZnTi_5_O_13_, Zn atoms were also found to occupy both tetrahedral and octahedral sites. Figure 8 shows the arrangement of idealized TiO_6_ and ZnO_6_ octahedra and ZnO_4_ tetrahedra in each level of *y* (= *n*/6, *n* = 1,2,3,…6) viewed along the *b*-axis. There is a substantially different arrangement at each level. No ZnO_4_ or ZnO_6_ units share polyhedral corners or edges.

##### 3.1.2.2 BaO-TiO_2_-Nb_2_O_5_ System

In the ternary BaO-TiO_2_-Nb_2_O_5_ system [63,64], a total of fourteen ternary compounds have been identified. Based upon compositions containing high (Ti+Nb)/Ba ratios, four of them were expected to have dielectric properties comparable to those of the polytitanates of barium. These are members of a chemically and structurally homologous series, AB_2_*_n_*_+1_O_4_*_n_*_+5_ with 3 < *n* < 6 with respective compositions of BaTi_3_Nb_4_O_17_, BaTi_5_Nb_4_O_21_, BaTi_7_Nb_4_O_25_, and BaTi_9_Nb_4_O_29_. The phase BaTiNb_4_O_13_ which could be considered as a *n* = 2 member of the chemical series, has a different type of structure, isomorphous to KTa_5_O_13_. It was also found that a small amount of Nb_2_O_5_ (mole fraction of 1 % to 5 %) is enough to change the stability for the close-packed layers of the Ba-polytitanate phases, Ba_6_Ti_7_O_40_ and Ba_4_Ti_13_O_30_ so that four new phases (having 8-layer orthorhombic, 20-layer orthorhombic, 7-layer mono-clinic, and 13-layer monoclinic lattices) form instead. All these barium niobium polytitanates have the 6-octahedra wide (≈ 17 Å) type unit cell. Detailed structure determination of some of the phases continues today.

#### 3.1.3 BaO-R_2_O_3_-TiO_2_ (R = lanthanides)

In the late 1970s and early 1980s, a composition close to 1:1:5 in the BaO:Nd_2_O_3_:TiO_2_ system was identified to having high dielectric constants (80 to 90), modest Q (3 to 4 K at 3 GHz), and near zero temperature coefficient of resonance frequency. Therefore dielectric ceramics based on this system have found important applications in modern electronic practices. The phase diagram of this system was determined by Kolar et al. [65]. Characterization of new phases in the BaO-R_2_O_3_-TiO_2_ systems has become important research activities since. For example, Olsen and Roth [69] determined the crystal structure of BaNd_2_Ti_3_O_10_ by electron diffraction and high-resolution electron microscopy to be monoclinic instead of the reported orthorhombic symmetry. The lower symmetry found was due to the octahedral tilt because of the relatively small Nd^3+^ cations. Crystal symmetry and lattice parameters of two other new phases, BaNd_2_Ti_3_O_10_, and BaNd_2_Ti_5_O_14_, have also been identified by single crystal growth [65].

In another study, the structure of the Ba_6−3_*_x_*Sm_8+2_*_x_*Ti_18_O_54_ solid solution was determined to be different from reported data due to the observation of superstructure reflections [70]. The structure, refined as orthorhombic *Pnma* and with a doubled *c*-axis (7.6(1) Å), is made up of a network of corner sharing TiO_6_^−2^ octahedra creating rhombic (perovskite-like) and pentagonal channels. While the two pentagonal channels are fully occupied by the Ba atoms, and one rhombic channel is fully occupied by Sm, in the remaining two rhombic channels, one is partially occupied by Sm and the other one is shared by Ba/Sm atoms.

### 3.2 Early 1990s to Present

Wireless communication has been a fast growing industry in recent years. Various applications include wireless FAX, cell phones, global position satellite (GPS), and direct broadcast satellites. Dielectric ceramics are used to make a variety of components in cellular communications circuits, such as resonators, oscillators, and bandpass filters for wireless communications. Relatively few ceramic systems are currently available with the properties needed for practical applications at various operating frequencies. For further miniaturization, new materials are still required.

Since the early nineties, the focus of the phase equilibrium studies of dielectrics at NIST has been on ceramic materials that are for wireless microwave communication applications. This microwave phase equilibria program is under the direction of Terrell Vanderah. Team members include R. S. Roth, I. Levin, B. Burton, E. Cockayne, W. Wong-Ng, J. Chan (now at the Chemistry Department of Louisiana State University), and A. Drews (now at the Ford Motor Research Laboratory). Collaborators at the NIST Neutron Reactor Division include B. Toby, A. Santoro, Q. Huang, and T. Amos. T. Siegrist of Lucent Technologies participates in determining single crystal structures. Since dielectric property is critical for determining the applications of these materials, property measurement is also an integral part of the program. Further collaboration also involves R. Geyer of NIST, Boulder, who conducts dielectric property measurements.

In addition to the studies of the titanate systems, research interest also extended to the tantalate and niobate systems. Up to today, representative oxide systems that have been pursued at NIST include BaO-Fe_2_O_3_-TiO_2_ [71], SrO-TiO_2_-Nb_2_O_5_ [72], SrO-Al_2_O_3_-Nb_2_O_5_ [73], CaO-Al_2_O_3_-Nb_2_O_5_ [74] and BaO-Al_2_O_3_-Nb_2_O_5_ [75], as well as work on CaO-SrO and BaO-Ta_2_O_5_ systems with and without TiO_2_. Of special note is the structure determination of the various polytypes of Ca_2_Ta_2_O_7_ [76].

#### 3.2.1 The BaO-Fe_2_O_3_-TiO_2_ System

**Fig. 9:**
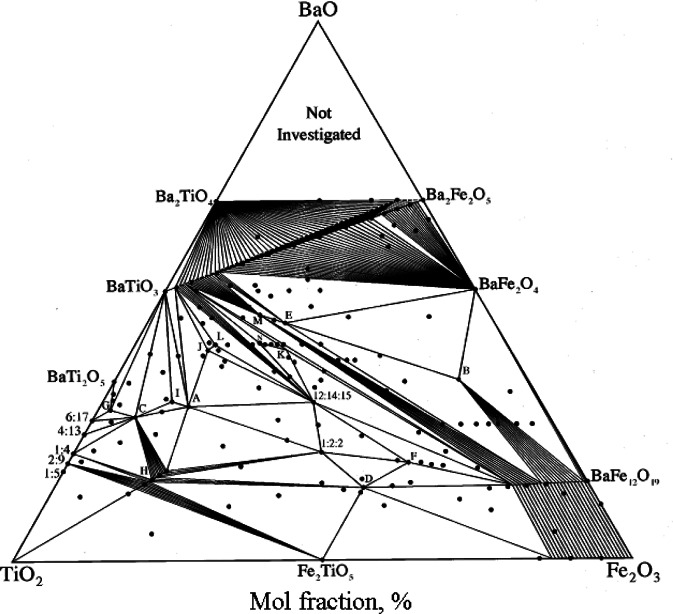
BaO-Fe_2_O_3_-TiO_2_ phase diagram (air, 1250 °C to 1270 °C) [72]. The compositions of the ternary phases found in the system are (BaO:Fe_2_O_3_:TiO_2_): A, 2:1:4; B,3:5:1; C,4:1:10; D,3:12:7; E, 4:2:3; F, 1:6:3; G, 14:1:35; H 1:1:5 and 1:1:4 (Hollandite); I, 8:3:16; J, 3:1:4; K, 8:5:8; L, 6:2:7; M, 8:3:6;, N, 2:1:2.

Ceramic magnetic oxides are essential components in a wide variety of electronic applications; for example, signal circulators and isolators. In the hope of obtaining improved materials that have higher dielectric constants while maintaining low dielectric losses and high saturation magnetization, phase equilibrium study of the BaO-Fe_2_O_3_-TiO_2_ system [71] was conducted to investigate the phase relations and crystal-chemical relations of magnetic iron-containing compounds with the technically important barium polytitanates. The phase relationships of this system was found to be very complex (Fig. 9). The overview of the binary systems and the phase relationships of the ternary system were discussed in detail. The system BaTiO_3_-Ba_2_Fe_2_O_5_ was found to contain tetravalent (Fe^+4^) ions when annealed at low temperatures and the oxidation/reduction and structural analysis was thoroughly investigated [77]. There are at least 16 ternary phases in the BaO-Fe_2_O_3_-TiO_2_ system, with 10 adopting new structure-types. Among them, crystal structure determination has been performed on the following phases: Ba_3_Fe_10_TiO_20_ [78], Ba_2_Fe_2_Ti_4_O_13_ [78], Ba_6_Fe_45_Ti_17_O_106_ [79], BaFe_11_Ti_3_O_23_ [79], Ba_4_Fe_2_Ti_10_O_27_ [78], Ba_11_Fe_8_Ti_9_O_41_ [80], and Ba_5_Fe_4_Ti_10_O_31_ [81]. Most of these phases exhibit layers of closed-packed structure built from [O,(Ba,O)] layers. One common feature of these structure is that the octahedral sites are mostly occupied by a mixture of Fe^3+^ and Ti^4+^ (occasionally with preferential ordering). Tetrahedral sites are occupied by Fe^3+^ only. Five examples of these structures are highlighted below.

##### 3.2.1.1 Ba_3_Fe_10_TiO_20_

**Fig. 10:**
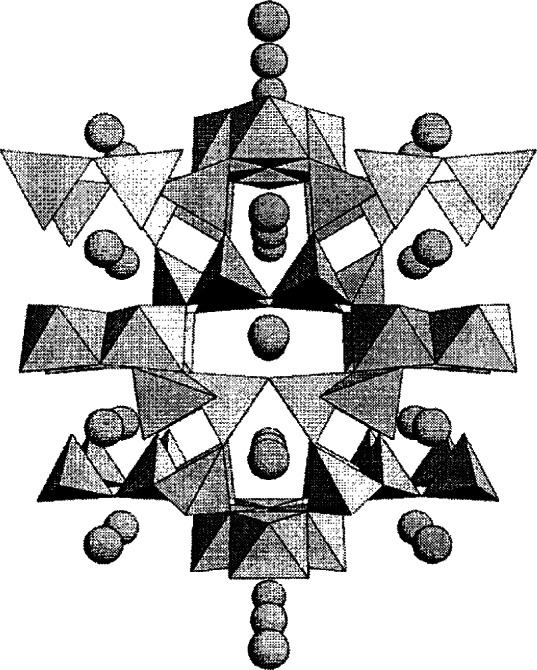
The monoclinic crystal structure of Ba_3_Fe_10_TiO_20_ (3:5:1) [78], perspective view approximately along the *c*-direction. Large spheres are Ba cations.

**Fig. 11:**
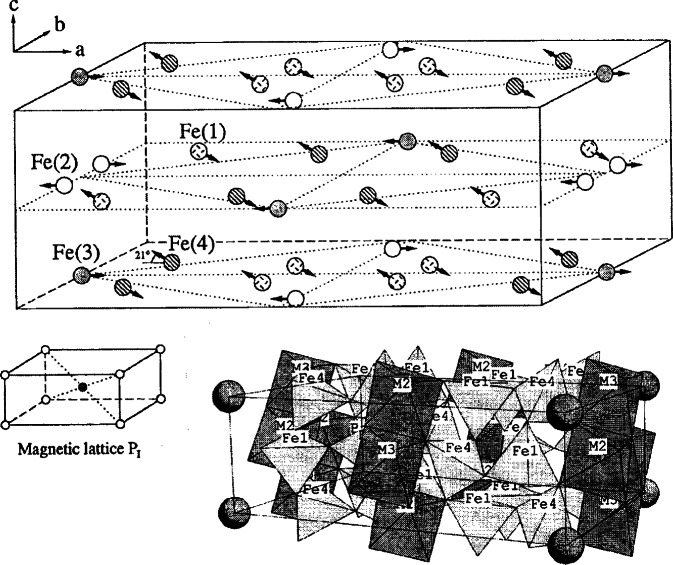
The magnetic structure model for Ba_3_Fe_10_TiO_20_ from room temperature structure study. The magnetic lattice exhibits *P*/2′ symmetry and is antibody-centered. The polyhedron linkage patterns among the magnetic sites are illustrated in the bottom part of the figure. Fe1 and Fe4 comprise one set of spins while M2 and M3 comprise the second set [78].

**Fig. 12:**
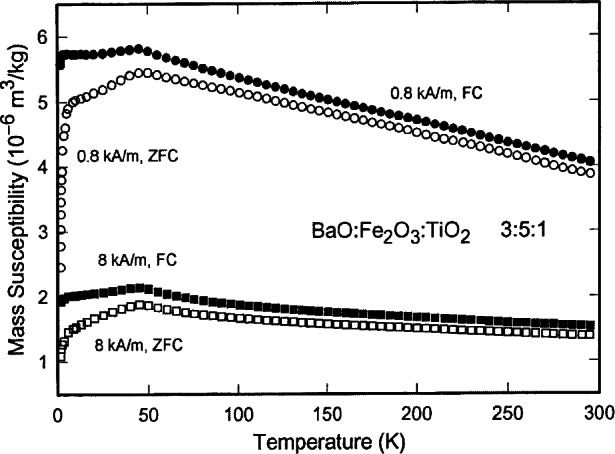
Magnetic susceptibility plot of Ba_3_Fe_10_TiO_20_ [78].

Ba_3_Fe_10_TiO_20_ has a relatively high Fe-content and adopts an open-framework type structure (Fig. 10) [78]. Both tetrahedral and octahedral coordination of the transition metals are featured. The rectangular and pentagonal channels are occupied by the barium ions. Ba_3_Fe_10_TiO_20_ (*I*2/*m*, *a* = 15.358(1) Å, *b* = 11.818(1) Å, *c* = 5.177(3) Å, and *β* = 91.231(4)° is isostructural with Ba_3_Fe_10_SnO_20_, Ba_3_Al_10_TiO_20_, Pb_3_Al_10_GeO_20_ and Pb_3_Al_10_SiO_20_ [78]. At room temperature, the compound has a magnetically ordered lattice with reduced symmetry and two antiferromagnetic sublattices. The polyhedral linkage among the magnetic sites is shown in Fig. 11. Fe1 and Fe4 comprise one set of collinear antiparallel spins in the vertex-linked tetrahedra. Magnetic interactions occur via superexchange. Another set of antiparallel spins are located in the edge-sharing Fe2-Fe3 octahedral sites. Direct interactions across shared edges are possible in addition to 90° superexchange via oxygen. The magnetic susceptibility plot as a function of temperature after zero field cooling and field cooling is shown in Fig. 12. Interactions among magnetic ions are substantial. These susceptibility curves indicate transitions at 5 K and 45 K. Data collected at 15 K, 6.5 K, and 1.8 K indicated that at least one other magnetic structure occurs below room temperature.

##### 3.2.1.2 Ba_6_Fe_45_Ti_17_O_106_ and BaFe_11_Ti_3_O_23_

**Fig. 13:**
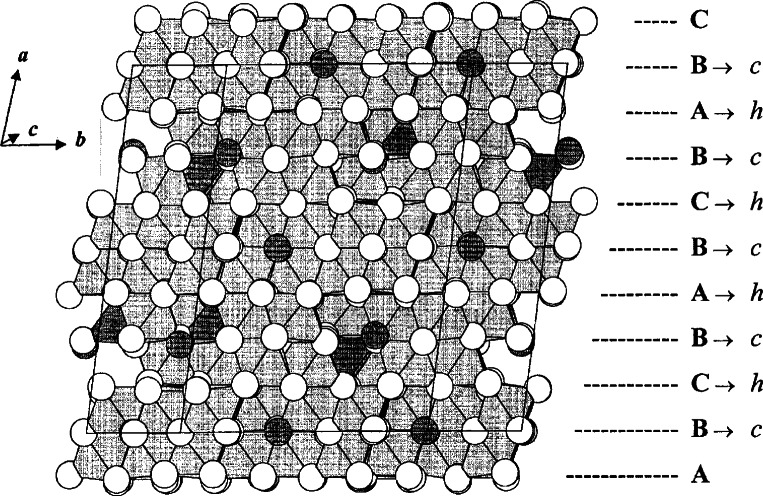
The structure of Ba_6_Fe_45_Ti_17_O_106_ as viewed approximately along the *c*-direction [79]. Successive layers alternate between *ccp* and *hcp* along the *a*-axis, resulting in an 8L structure with a (*ch*)_4_ repeat pattern. Shaded spheres denote Ba ions.

The overall structure of Ba_6_Fe_45_Ti_17_O_106_ (*C*2/*m*, *a* = 19.390(1) Å, *b* = 20.260(1) Å, *c* = 10.076(1) Å, *β* = 105.27(1)°) was solved with the single crystal diffraction method, and the exact stoichiometry was studied by neutron Rietveld refinements [79]. This compound exhibits variable stoichiometry, Ba_6_Fe_48−_*_x_*Ti_14+_*_x_*O_106_, with Fe:Ti ratio dependent upon the partial pressure of oxygen. The *x* value is 3 when prepared in air, and approaches ‘0’ when prepared in 100 % O_2_. It is a partially reduced phase with three Fe^2+^ per formula. The structure of Ba_6_Fe_45_Ti_17_O_106_ as viewed approximately along the *c*-axis, drawn to emphasize the stacking sequence of the close-packed [O,(Ba,O)] layers, is shown in Fig. 13. This eight-layer close-packed structure is built from alternating *ccp* and *hcp* layers [stacking sequence (*ch*)_4_] along the *a*-direction. Despite the high Fe content, it is a weak magnetic compound due to antiferromagnetic-type interactions.

**Fig. 14:**
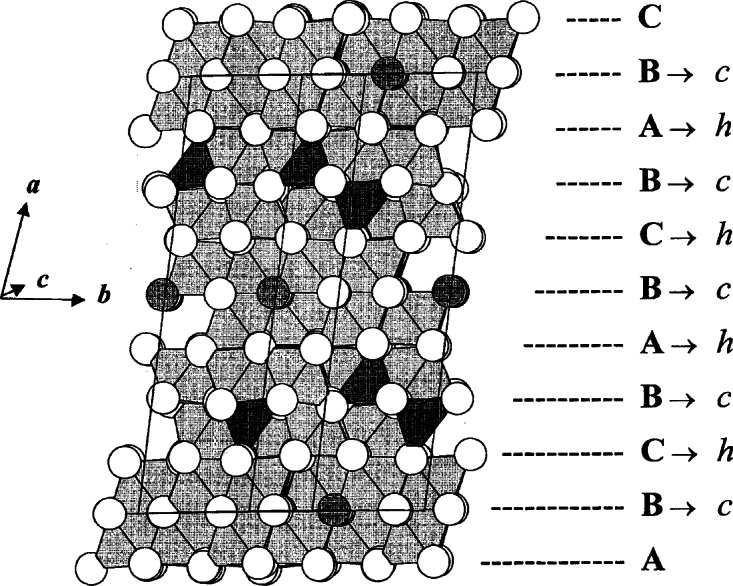
The structure of BaFe_11_Ti_3_O_23_ as viewed approximately along the *c*-direction emphasizing the stacking sequence of the close-packed [O, (Ba,O)] layers [79]. The resulting (*ch*)4 repeat pattern is the same as in Ba_6_Fe_45_Ti_17_O_106_. Shaded spheres denote Ba ions.

**Fig. 15:**
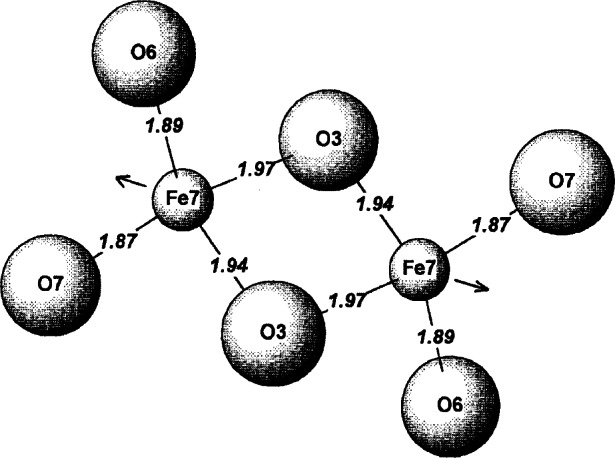
Local coordination about the tetrahedral Fe7 sites in the BaFe_11_Ti_3_O_23_ structure, with bond distances indicated [79].

During the crystal growth experiments of Ba_6_Fe_45_Ti_17_O_106_, a co-product crystal was also obtained, which is metastable in air (could not be prepared as a polycrystalline sample when cooled from above the solidus). The structure of this phase, BaFe_11_Ti_3_O_23_ (*C*2/*c*, *a* = 19.561(1) Å, *b* = 8.6614(7) Å, *c* = 10.120(1) Å, *β* = 105.62(1)°) was found to be related to but simpler than that of Ba_6_Fe_45_Ti_17_O_106_ (Fig. 14). It has the same stacking sequence, and has exactly the same number of layers as that of Ba_6_Fe_45_Ti_17_O_106_, but with a smaller *b*-dimension. This compound is also partially reduced, with 1 mol of Fe^2+^ per formula unit. The structure has some interesting features that may relate to its metastable nature. It has pairs of very unusual edge-sharing tetrahedra (Fig. 15). The expected cation-cation repulsion effects are observed in elongated Fe7-O3 distances with Fe displaced away from the common tetrahedral O3-O3 edge. Four out of eight close-pack layers in the structure are Ba-free.

##### 3.2.1.3 Ba_11_Fe_8_Ti_9_O_41_

**Fig. 16:**
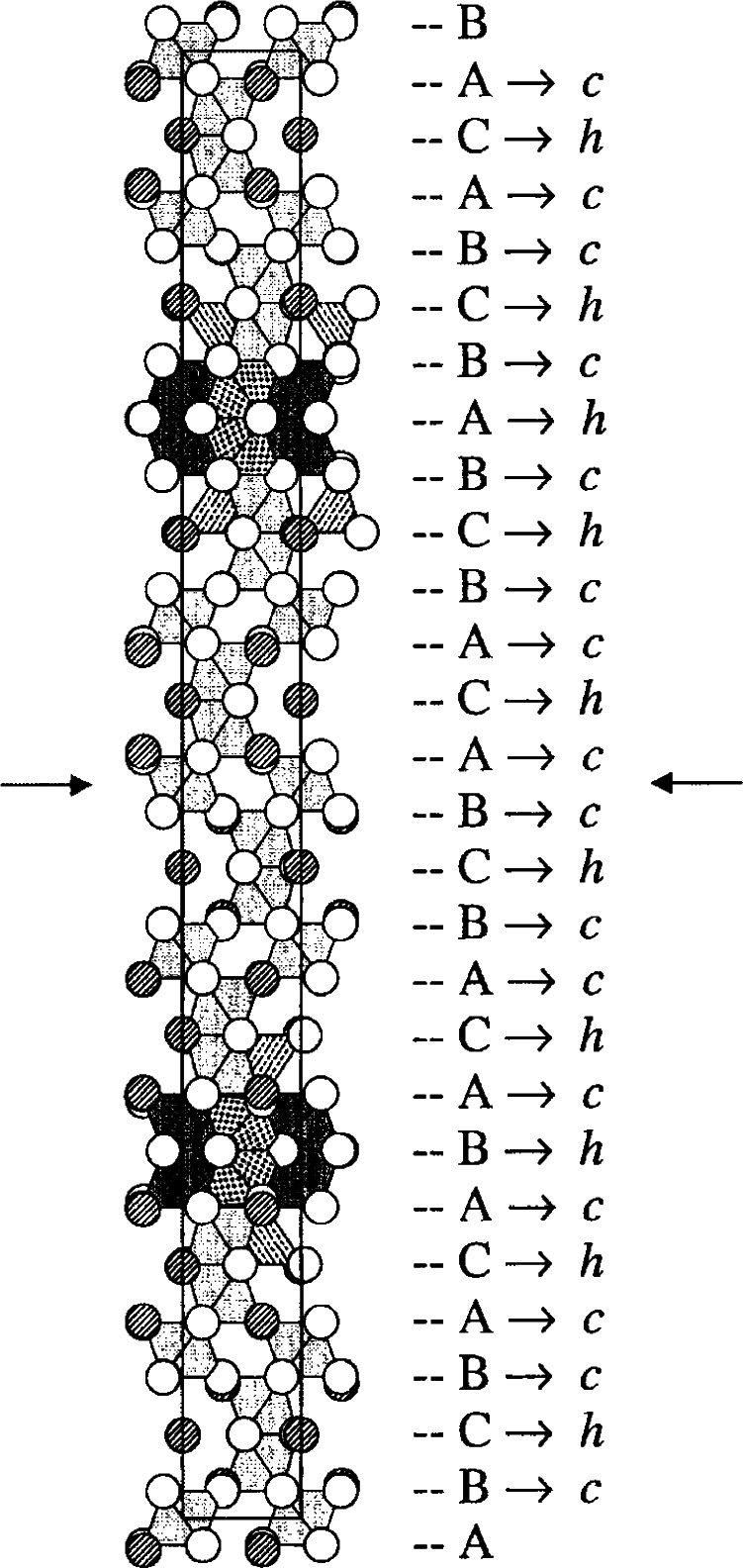
The hexagonal structure of Ba_11_Fe_8_Ti_9_O_41_ as viewed approximately along the 110 direction to emphasize the close-packed [O, (Ba,O)] layers [80]. The 26-layer structure of this compound exhibits the stacking sequence (*chcchchchcchc*)_2_. White spheres represent oxygen, striped spheres barium, and the light gray octahedra are preferrentially occupied by Ti. The darker gray and stippled octahedra occur as face-sharing pairs and are preferentially occupied by Fe. The hatched tetrahedra represent the Fe1 sites. Thus, the magnetic Fe atoms concentrate within four contiguous *cp* layers around *z* = ¼ and ¾.

This phase has an exceptionally long *c*-axis (*P*6_3_/*mmc*, *a* = 5.7506(3) Å, *c* = 61.413(2) Å) and therefore presented a crystallographic challenge [80]. The structure consists of 26 close-packed [O,(Ba,O)] layers (Fig. 16) with a stacking sequence (*chcchchchcchc*)_2_. The magnetic Fe ions were found to concentrate within four contiguous *cp* layers around *z* = ¼ and ¾. Unusual structural features, including cation disorder associated with unreasonably short cation-cation separations, were observed to occur within magnetic sections of the structure. This portion of the structure has been referred to as ‘natural magnetic multilayers’. Preferential ordering of Fe and Ti were found in the octahedral sites.

##### 3.2.1.4 Ba_5_Fe_4_Ti_10_O_31_

**Fig. 17:**
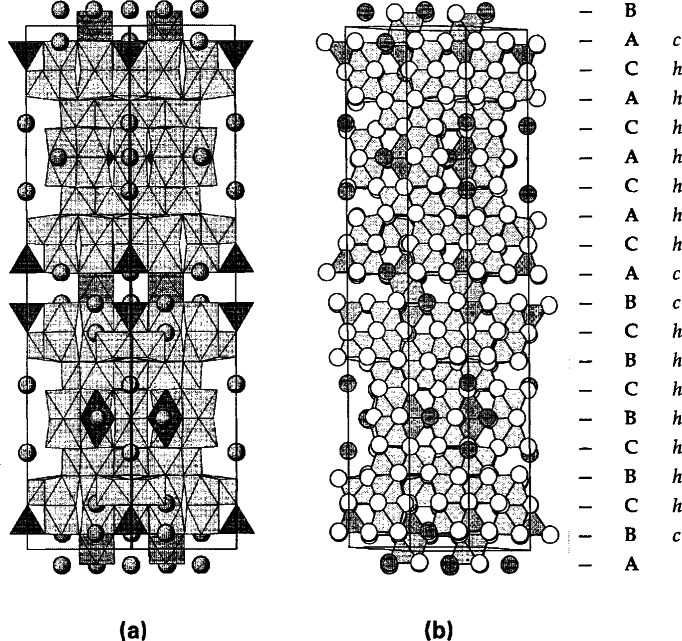
Views of the 18L structure of Ba_5_Fe_4_Ti_10_O_31_ along directions parallel to the close-packed [O,(Ba,O)] layers. (a) 〈110〉 view emphasizing polyhedral arrangements, oxygen atoms are denoted by vertices, barium as spheres. (b) 〈100〉 view with oxygens drawn as white spheres, Ba as gray, highlighting the close-packed layer sequence. The stacking pattern results in an overall (*chhhhhhhc*)_2_ close-packed structure.

This phase also has a relatively long *c*-axis (*P*6_3_/*mcm*, *a* = 9.9886(2) Å, *c* = 42.226(2) Å; *Z* = 6). It crystallizes in 18-layer close-packed structure built from vacancy-free [O,(Ba,O)] layers (Fig. 17) [81]. Preferential ordering of Fe^3+^ and Ti^4+^ was found in the octahedral sites. The tetrahedral sites are occupied by Fe^3+^, and one of which shares faces and is half occupied. Some Ba ions in the structure displays (9 + 3) coordination with three unusually long Ba-O bond distances. The structure contains residual strain not relieved by distortion. The compound exhibits approximately paramagnetic behavior with small deviations from the Curie Law. The relative permittivity and dielectric loss tangent values (measured between 7.1 GHz and 7.7 GHz) were 32 × 10^−3^ and 3.3 × 10^−3^, respectively.

#### 3.2.2 The SrO-Nb_2_O_5_-TiO_2_ System

The cubic SrTiO_3_ phase has a high dielectric constant but a negative temperature coefficient. This is a potentially useful microwave ceramic that requires second-phase compensation. During the phase equilibria studies, more than 15 ternary compounds have been confirmed. Documentation of the phase diagram of this system is currently in progress [72]. The crystal structure of various homologous series have been investigated in this laboratory. Three such series will be highlighted. Two series feature perovskite slabs while the third one consists of rutile slabs.

**Fig. 18:**
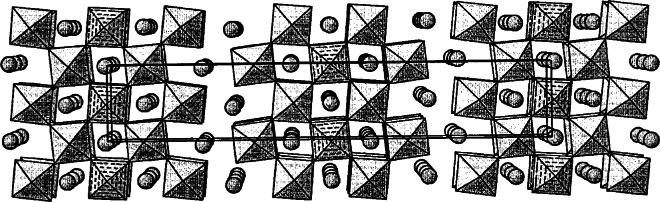
Perspective view of the structure of Sr_5_TiNb_4_O_17_ approximately along *c*. Sr atoms are shown as spheres. Hatchedoctahedra are preferrentially occupied Ti [82]. Oxygen sites are at the vertices of the octahedra.

The first perovskite homologous series can be described with the formula of A*_n_*B*_n_*O_3_*_n_*_+2_ (A = Sr; B = Ti, Nb). The structure of the *n* = 5 member (Fig. 18) [82] consists of alternating perovskite slabs built from five layers of distorted octahedra. Each slab is offset with respect to each other by *a*/2 and *c*/2. All atoms are found to be on the mirror planes at *z* = 0 and 1/2. Nb^5+^ was found to be associated with highly distorted octahedra near the gap of the block, whereas the largest amount of Ti^4+^ is found in the center of the slabs, where octahedral distortion is the least.

**Fig. 19:**
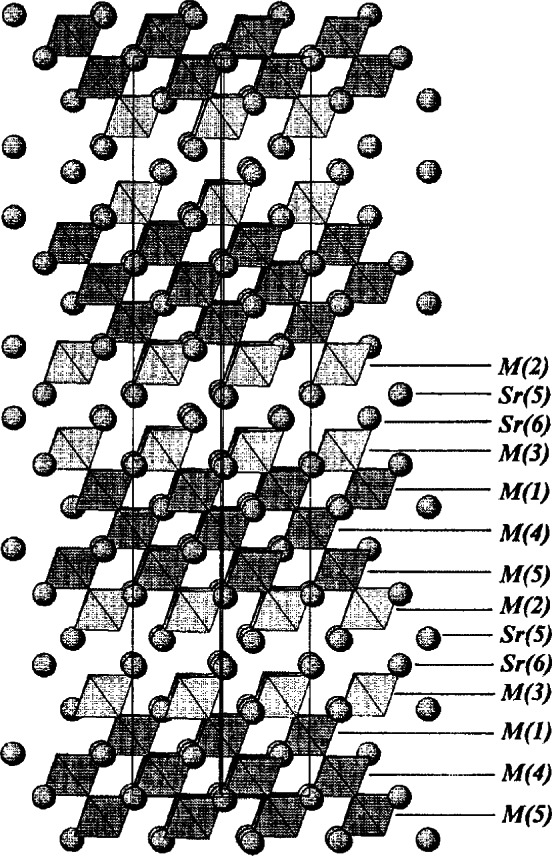
Crystal structure of Sr_6_TiNb_4_O_18_ viewed along the 〈110〉 direction [83]. Sr atoms are shown as spheres.

The compounds of the perovskite homologous series A*_n_*B*_n_*_−1_O_3_*_n_* have a rhombohedral structure, and the thickness of the slab is (*n* − 1) units of octahedra [83]. The crystal structure of the *n* = 6 member viewed along the 〈110〉 direction is shown in Fig. 19. It consists of infinite perovskite-type slabs with five octahedra in thickness. Sr atoms are shown as spheres. Ti^4+^ and Nb^5+^ are distributed among the octahedral sites. All Sr ions are 12-coordinated. Similar to the previous perovskite series, octahedral sites have mixed Nb^5+^ and Ti^4+^ ions with preferential ordering of Nb^5+^ at the edge of the slabs adjacent to the gap.

**Fig. 20:**
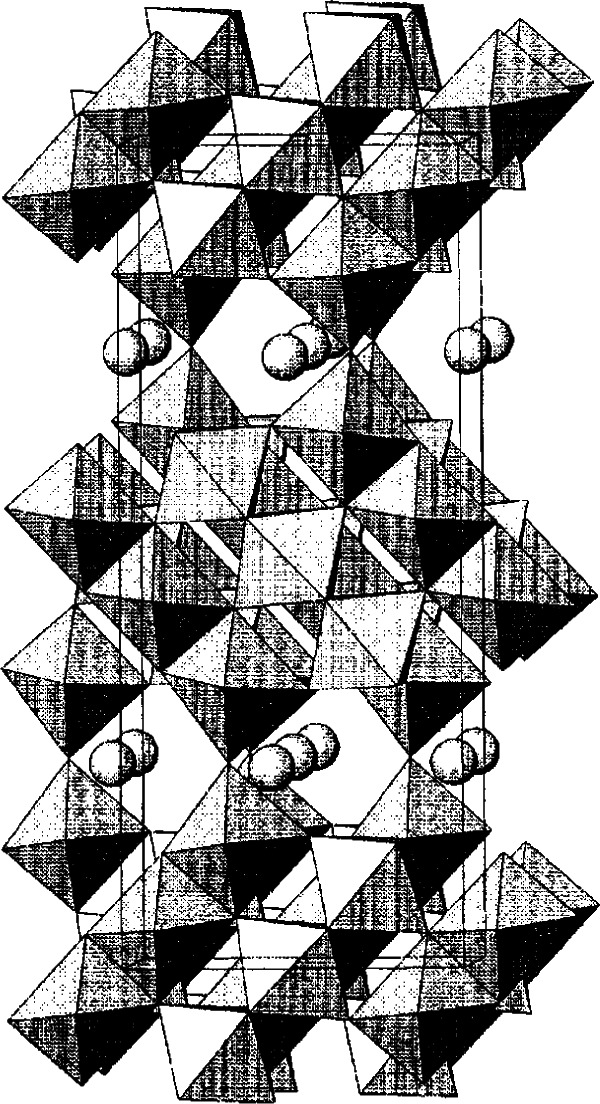
The orthorhombic structure of SrTi_3_Nb_4_O_17_, the *n* = 3 member of the “chemically twinned rutile oxides”, SrM_2_*_n_*_+1_O_4_*_n_*_+5_ series, viewed approximately along the 〈100〉 direction [84]. Spheres represent Sr^2+^ ions.

The *c* parameters of seven successive members of the homologous rutile series SrM_2_*_n_*_+1_O_4_*_n_*_+5_ were found to increase from about 21 Å to 47 Å by approximately 4.4 Å for each additional value of n [84]. This increase corresponds to adding two TiO_2_ units to the width of the rutile slab. In this series, all phases essentially have similar dimension of *a* and *b*. In the structure, blocks of rutile are joined together by vertex-sharing octahedra. Adjacent rutile slabs are related by a mirror plane containing the Sr ions, therefore it can be viewed as a “chemically twinned rutile” oxide. Figure 20 illustrates the structure of the *n* = 3 member, viewed approximately along the 〈100〉 direction. Mixed Nb and Ti were found in these octahedral sites. Similar to the two perovskite series, Nb^5+^ was also found to preferentially occupy the highly distorted octahedral sites at the border of the slabs.

#### 3.2.3 AO-Al_2_O_3_-Nb_2_O_5_ (A = Ca, Sr, and Ba)

Currently Ba_3_MTa_2_O_9_, with M = Zn or Mg are microwave materials that are used by industry for high-frequency, high-power resonator applications because of their unique dielectric properties (high permittivities (about 30), and also with low dielectric loss tangents (< 10^−5^ at 1 GHz)). The drawback of these materials, however, is their costly production. The ternary AO-Al_2_O_3_-Nb_2_O_5_ systems were studied because the polarizability of Ta^5+^ is intermediate between Al^3+^ and Nb^5+^, and Nb_2_O_5_ is much less expensive then Ta_2_O_5_.

**Fig. 21:**
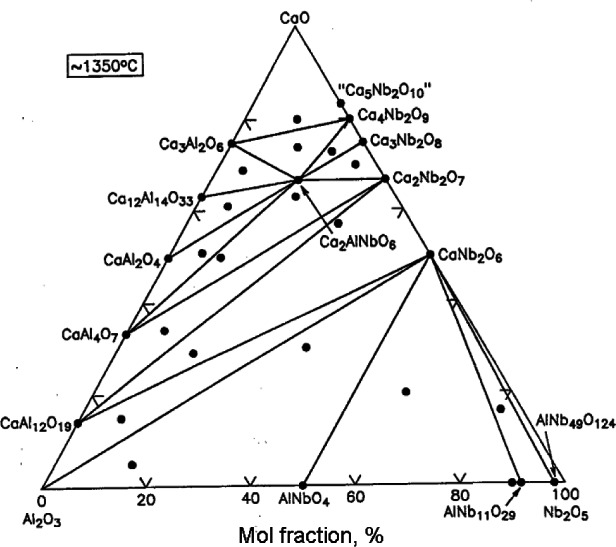
Subsolidus phase equlibria relations found in the CaO:Al_2_O_3_-Nb_2_O_5_ system in air [73].

Figure 21 shows the phase diagram of the CaO-Al_2_O_3_-Nb_2_O_5_ system [74]. Only one ternary compound was found, namely, Ca_2_AlNbO_6_, which is a perovskite derivative. The structure of this phase is similar to that of the othorhombic CaTiO_3_, except with Al^3+^ and Nb^5+^ ordered in a 1:1 manner. The slight distortion of the cell gives rise to the monoclinic space group *P*2_1_/*n* (*a* = 5.3780(1) Å, *b* = 5.4154(1) Å, *c* = 7.6248(2) Å, and *β* = 89.968 (2)°. A significant finding of this study pertains to the dielectric properties of compositions prepared between Ca_3_Nb_2_O_8_ and Ca_2_AlNbO_6_. These compositions have relatively high dielectric constants and high *Q*; and above all, can be tuned to having near-zero temperature coefficient. Theoretical calculations are being pursued by B. Burton and E. Cockayne to understand and predict the microwave properties of these materials.

**Fig. 22:**
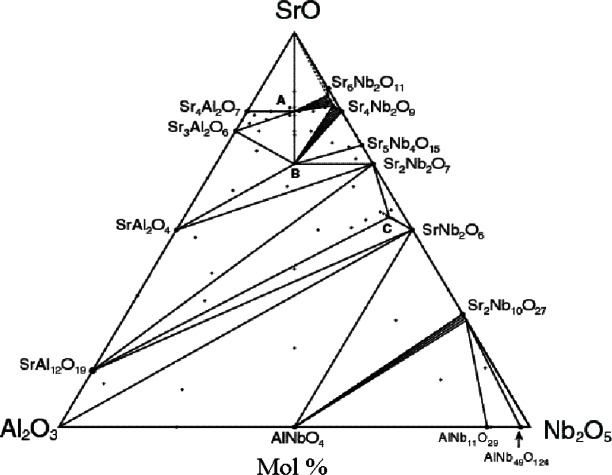
Subsoldius phase equilibria relations in the SrO-Al_2_O_3_-Nb_2_O_5_ system determined in air with synthesis temperatures 1200 °C to 1600 °C [74].

The phase diagram of the SrO-Al_2_O_3_-Nb_2_O_5_ system is shown in Fig. 22. In this system, in addition to the double-perovskite phase, Sr_2_AlNbO_6_ (*Fm*3*m*, *a* = 7.7791(1)), two new ternary compounds, Sr_4_AlNbO_8_ and Sr_5.7_Al_0.7_Nb_9.5_O_30_ were also discovered. Sr_5.7_Al_0.7_Nb_9.5_O_30_ adopts the tetragonal tungsten bronze (TTB) type structure. The general formula can be written as Sr_6−_*_x_*Al_1_ *_x_*Nb_9+_*_x_*O_30_. A narrow stability region is observed, and the TTB structure forms only at *x* = 0.3, in the high temperature range of 1375 °C to 1425 °C. This is a solid solution with Al and Nb mixed within the octahedra. Electron diffraction results indicated that the strontium-rich Sr_4_AlNbO_8_ phase crystallizes with the monoclinic *P*2_1_/*c* symmetry.

**Fig. 23:**
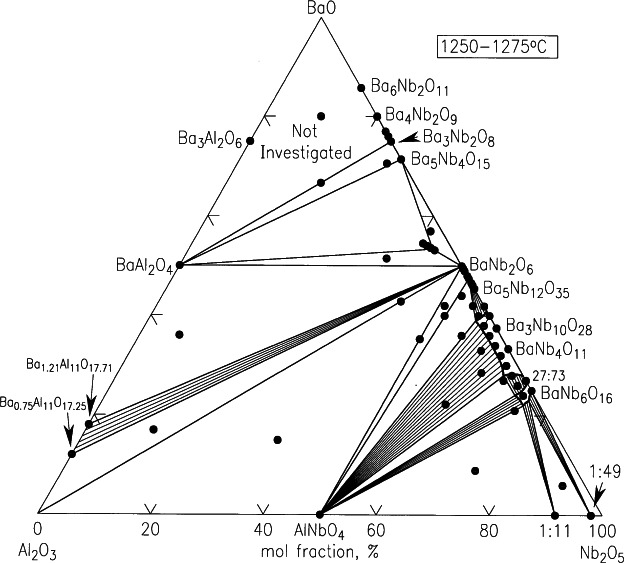
Subsolidus phase equilibria relations in the BaO-Al_2_O_3_-Nb_2_O_5_ system as determined in air. The region above the BaAl_2_O_4_-Ba_3_Nb_2_O_8_ tie-line was highly reactive with moisture and CO_2_, and was not investigated further [75].

**Fig. 24:**
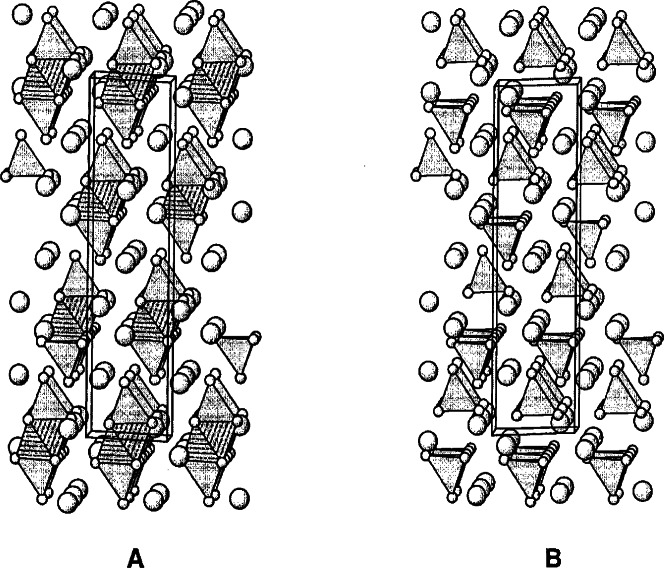
Structures proposed for Ba_3_Nb_2_O_8_; (A) from analysis of x-ray powder diffraction data [85], and (B) from single crystal study [75]. Large spheres represent Ba ions, smaller spheres are oxygens, and Nb ions occupy the polyhedra. The models are in agreement except that as found in (B), the [NbO_6_] sites were empty and that all Nb ions occupy discrete tetrahedral sites.

In the BaO-Al_2_O_3_-Nb_2_O_5_ system (Fig. 23) [75], only one ternary phase was found: Ba_5.75_Al_0.75_Nb_9.25_O_30_ (Ba_6−_*_x_*Al_1_ *_x_*Nb_9+_*_x_*O_30_, *x* = 0.25), which crystallizes with a tetragonal tungsten bronze (TTB) type structure (*P*4*bm*; *a* = 12.558(1), and *c* = 3.9708(3) Å). This phase exhibited an ambient dielectric constant of 242, no indication of ferroelectric behavior was observed in the temperature range 100 K to 400 K. The subsystem BaO-Nb_2_O_5_ has been studied extensively because of its complex nature. The crystal chemistry of this binary system can be largely described by octahedral units [NbO_6_] except for Ba_3_Nb_2_O_8_, which was reported (using powder x-ray method) to adopt the salt-like palmierite-type structure with isolated tetrahedral [NbO_4_] units [85]. Single crystal structure study of this highly unstable phase in our laboratory, while confirming the tetrahedral environment of Nb (Fig. 24), found that all octahedral sites around Nb are empty [75]. This finding is contrary to the previous report of a partial 15 % occupancy. All Nb metals occupy discrete tetrahedra, which is unusual and may explain its instability in the presence of water and air.

## 4. Phase Equilibria and Crystallographic Studies of High-Temperature Superconductors (1986 to Present)

Investigation of high temperature superconductor materials has been an exciting and challenging undertaking in the past fifteen years. Since 1986, a large number of multicomponent high-temperature superconductor oxide materials have been discovered; most of these compounds exhibit complex chemistry, including non-stoichiometry, defects and incommensuracy. It was recognized early on that phase equilibrium information is critical to the processing of the high *T*_c_ materials. Scientists from the Ceramics Division of NIST have since been involved with the programs of the Electric Power Research Institute (EPRI) and Department of Energy (DOE) in conducting phase equilibria research. External collaborations include Oak Ridge National Laboratory, Argonne National laboratory, Los Alamos National Laboratory, Princeton University, Florida State University, State University of New York (Buffalo), Bell Telephone Laboratory, BP-Amoco Research Center, and the Airforce Research Laboratory. Collaborators from other divisions of NIST include B. Toby, Q. Huang, A. Santoro, and J. Stalick of the Nuclear Reactor Division; C. K. Chiang of the Polymer Division; A. Kearsley of the Mathematical and Computational Sciences Division; R. Shull, L. H. Bennett, and L. Swartzendruber from Metallurgy Division; J. Suh, J. Dillingham, W. Greenwood, R. Coutts, F. Jiang and G. Thielz are student assistants from University of Maryland.

In addition to more than 150 combined journal, review articles, and proceeding papers related to phase equilibria and crystallography of high *T*_c_ related phases from the Ceramics Division, various books and book chapters have also been published. For example, T. A. Vanderah, J. D. Whitler, R. S. Roth, and H. F. McMurdie have compiled two volumes of Phase Diagrams for High *T*_c_-Superconductors [86–87]. D. L. Kaiser and L. F. Schneemeyer (Lucent Technologies) have co-authored a handbook chapter on “Single Crystal Growth” [88]. Wong-Ng has authored a handbook chapter on “Phase Diagrams” [89], and other book chapters on “Structure of Bi-containing materials” [90], and on “Superconductors: Structures and Applications” [91]. She has also co-authored with S. W. Freiman on “Superconducting Phase Formation in Bi(Pb)-Sr-Ca-Cu-O Glass: A Review” [92].

More than 150 reference x-ray diffraction patterns of high *T*_c_ and related phases have been prepared at NIST and published in the ICDD Powder Diffraction File (PDF) [1]. A large portion of these patterns are results of collaborations with outside organizations such as BP-Amoco Research (James A. Kaduk), North Dakota State University (Gregory J. McCarthy), and Georgia Institute of Technology (R. A. Young).

### 4.1 Ba-Y-Cu-O Systems

#### 4.1.1 Subsolidus Phase Diagrams of BaO-Y_2_O_3_-CuO

**Fig. 25:**
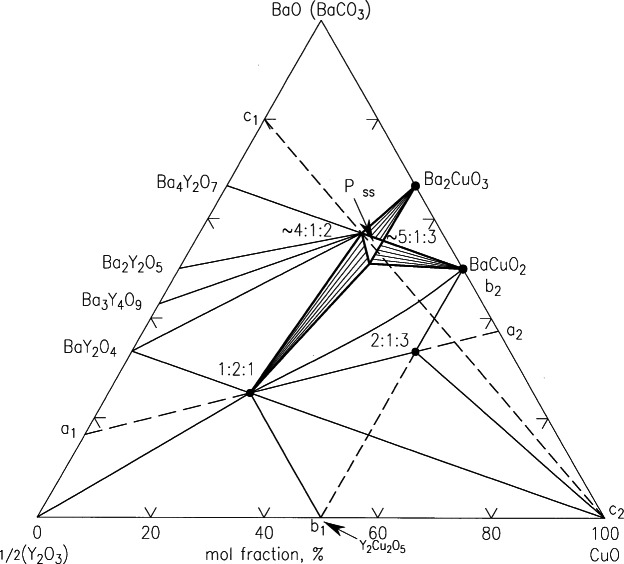
Phase diagram of the ternary system BaO(BaCO_3_)-½Y_2_O_3_-CuO at ≈ 950 °C [93]. The position of the Ba_2_YCu_3_O_6+_*_x_* (2:1:3) superconductor and the green phase BaY_2_CuO_5_ (1:2:1) are shown.

Roth et al. [93] were among the first to determine a complete phase diagram of the BaO(BaCO_3_)-Y_2_O_3_-CuO system, at ≈950 °C (Fig. 25). This diagram has been used extensively as a primary reference for many years. The presence of CO_2_ has a substantial influence on the phase formation in the Ba-rich region. Four phases were observed in the BaO(BaCO_3_)-2(Y_2_O_3_) subsystem. Barium rich Ba_4_Y_2_O_7_ and Ba_2_Y_2_O_5_ have been determined to be oxycarbonates, with formulas of Ba_4_Y_2_O_7_CO_2_ and Ba_2_Y_2_O_5_2CO_2_. When pure BaO is used instead of BaCO_3_, the Ba_4_Y_2_O_7_ and Ba_2_Y_2_O_5_ compounds can not be prepared in the absence of CO_2_ in the atmosphere. There are a total of 3 ternary oxides, including the high temperature superconductor Ba_2_YCu_3_O_6+_*_x_*, (2:1:3), and an impurity phase BaY_2_CuO_5_ (1:2:1) which is known as the “green phase”. Another barium rich oxycarbonate solid solution region is known as “the other perovskite phase”, and is bounded by the 4:1:2, 5:1:3 and 3:1:2 compositions. The Ba_2_YCu_3_O_6+_*_x_* phase is known to exhibit an orthorhombic-tetragonal phase transition depending on the oxygen content [94].

**Fig. 26:**
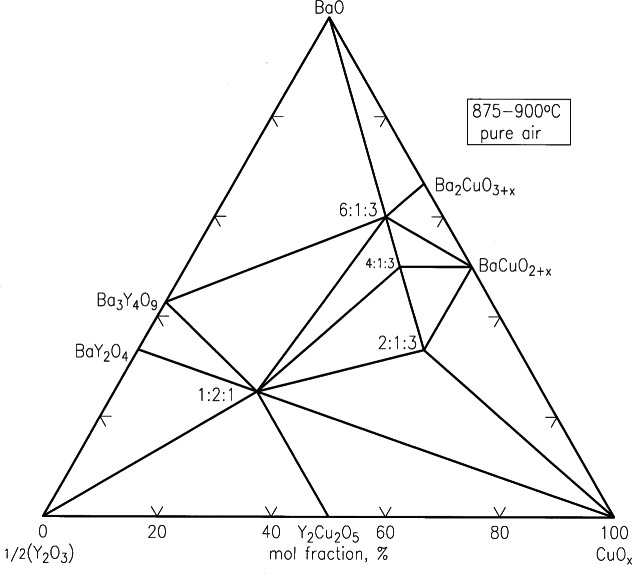
Phase diagram of the BaO-½Y_2_O_3_-CuO*_x_* system prepared at *p*_O2_ = 21 kPa (875 °C to 900 °C). In this diagram, the symbols (2:1:3), (4:1:3), (6:1:3), and (1:2:1) are used to represent the phases BaY_2_CuO_5_, Ba_4_YCu_3_O*_x_*, Ba_6_YCu_3_O*_x_*, and BaY_2_CuO_5_, respectively [94].

**Fig. 27:**
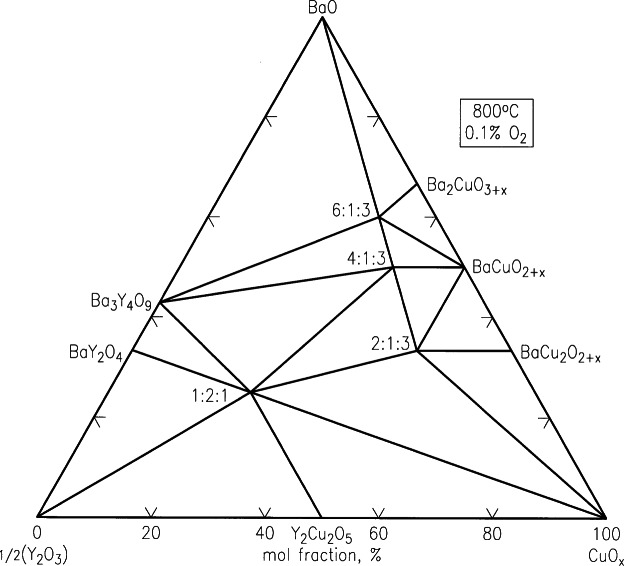
Phase diagram of the BaO-½Y_2_O_3_-CuO*_x_* system prepared at *p*_O2_ = 100 Pa (800 °C to 810 °C). Symbols are shown in Fig. 2 [94].

Recently, second-generation high *T*_c_ superconductor tapes based on Ba-R-Cu-O (R = Y and lanthanides) materials deposited on flexible coated-conductors have received considerable attentions [95,96]. The Ba-R-Cu-O materials are relatively more isotropic when compared with Bi(Pb)-Sr-Ca-Cu-O (BSCCO)-based superconductors, and can retain current carrying ability at liquid nitrogen temperature under high magnetic fields. Because of the potential advantages of coated-conductor superconductors, a renewed research interest in the entire family of the Ba_2_RCu_3_O*_x_* materials has been developed. Processing of Ba_2_RCu_3_O*_x_* is typically carried out with carbonate-free precursors, phase equilibrium studies under carbonate-free and moisture-free conditions are necessary. Special apparatus and procedures for handling these atmospherically sensitive compositions have been developed. Wong-Ng and Cook [97] have successfully investigated the phase diagrams of the BaO-Y_2_O_3_-CuO*_x_* system at two oxygen partial pressures using carbonate-free precursors based on BaO. Experimental results were used to establish tie-lines in the BaO-rich part of the phase diagram (Figs. 26 and 27), and have confirmed a difference in tie-line distribution among the Ba_2_YCu_3_O*_x_*, Ba_4_YCu_3_O*_x_*, BaY_2_CuO_5_, and BaCuO_2+_*_x_* phases under carbonate-free conditions relative to those obtained using BaCO_3_-derived starting materials. By reducing *P*_O2_ from 21 kPa to 100 Pa under carbonate-free conditions, an additional tie-line change among the phases Ba_2_YCu_3_O*_x_*, Ba_4_YCu_3_O*_x_*, Ba_6_YCu_3_O*_x_* and BaY_2_CuO_5_ was observed.

#### 4.1.2 Thermomechanical Detwinning of Superconducting Ba_2_YCu_3_O_7−_*_x_* Single Crystals

**Fig. 28:**
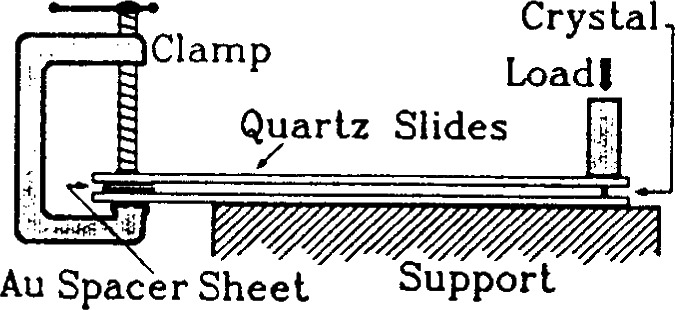
Schematic diagram of the experimental apparatus used to detwin YBCO single crystals [98].

Ba_2_YCu_3_O_7−_*_x_* is structurally similar to ferroelastic crystals which exist in two or more states, of which a selected state is favored during the application of a mechanical stress. The transformation from one orientation to another in BYC only requires a small atomic shift and oxygen ordering. In order to obtain “true” single crystals, D. L. Kaiser and F. W. Gayle have developed an ingenious method for the complete removal of twins from Ba_2_YCu_3_O_6+_*_x_* single crystals by applying a uniaxial compressive stress along an *a*/*b* axis at elevated temperature [98]. Figure 28 shows the schematic diagram of the experimental apparatus used to detwin the Ba_2_YCu_3_O_6+_*_x_* single crystals. Two fused-quartz slides coated with evaporated gold are held in a parallel geometry by a clamp. The spacing between the slides is established by a thickness of gold filler sheets. An aluminum crucible filled with zirconia powder was used to apply the load to the standing crystal (grown from an Ba-Y-Cu-O melt in a gold crucible) on the edge (with the *c*-axis in the plane of the slide). The rig was then put inside a box furnace and heated in air to 600° C (below transformation temperature) to avoid surface roughness.

The twin-free crystals proved invaluable both for confirming or refining the crystal structure and for measuring anisotropy of physical properties along the *a* and *b*-directions in the Cu-O basal plane. In collaboration with S. F. Watkins and F. Fronczek of Louisiana State University [99], single crystal structure determination of a twin-free crystal was completed. Oxygen positions and occupancies in the Cu-O basal plane have been refined, showing that while the basal plane oxygen site in the *a*-direction is completely vacant, the oxygen atoms in *b*-direction are offset from the crystallographic mirror plane positions by 0.15 Å in a zig-zag fashion. A 2 % mass fraction Au was found to occupy the chain Cu sites. Weak superlattice reflections suggest a possible 3-dimensional ordering of O and/or Au.

#### 4.1.3 Liquidus Diagrams of the Ba-Y-Cu-O System

**Fig. 29:**
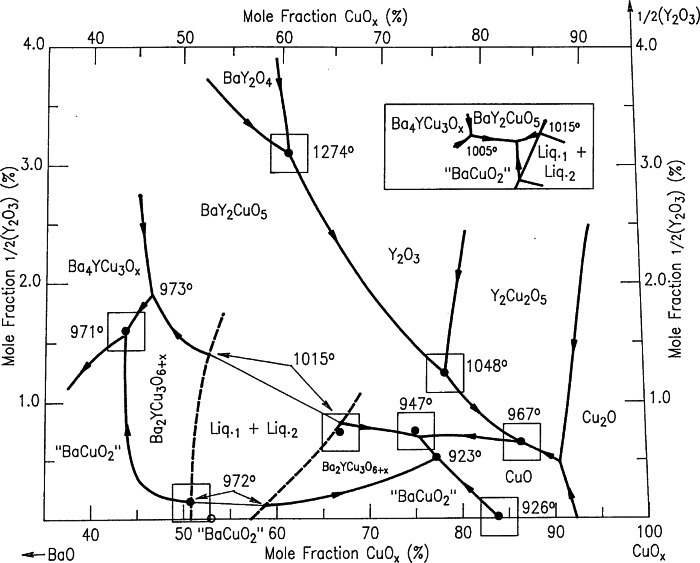
The Ba-Y-Cu-O liquidus showing the primary phase fields adjacent to the Ba_2_YCu_3_O_6+_*_x_* primary field, as defined by invariant (in air) melt compositions. The inset shows schematically the phase relations at the BaO-rich termination of the Ba_2_YCu_3_O_6+_*_x_* field when BaCO_3_, rather than BaO, was used to prepare the starting materials [100].

Melt processing investigations of Ba_2_YCu_3_O_6+_*_x_* for viable commercial applications constitute a major activity within the high *T*_c_ superconductor research community. The liquidus information for the Ba-Y-Cu-O system (the primary phase field for Ba_2_YCu_3_O_6+_*_x_*, and the univariant reactions in the phase diagram near the CuO-rich corner) is critical for crystal growth and melt processing. Our studies provided evidence that the liquid field of the BYC phase has a miscibility gap, as shown in Fig. 29 [100]. This diagram is presented by “stretching” the customary ternary composition triangle in order to magnify the yttrium oxide contents of the liquids, all of which were below 4 % (mole fraction). The phase fields of BaY_2_O_4_, Y_2_O_3_, BaY_2_CuO_5_, Ba_4_YCu_3_O*_x_*, Y_2_Cu_2_O_5_, Cu_2_O, nominal BaCuO_2_, and CuO are also shown. The crystallization field of the Ba_2_YCu_3_O_6+_*_x_* phase occurs in two segments. Both segments of the field are entirely below the 2.0 mol fraction % ½Y_2_O_3_ level. The right hand segment of the Ba_2_YCu_3_O_6+_*_x_* field is bounded by the crystallization fields of BaY_2_CuO_5_, “BaCuO_2_”, Y_2_Cu_2_O_5_ and CuO, and the left hand segment was bounded by the Ba_4_YCu_3_O*_x_*, BaY_2_CuO_5_ and “BaCuO_2_” primary phase fields. Two immiscible liquids were found. The determination of the Ba-Y-Cu-O (213) crystallization field shows an extension into the Ba-rich region, which opens up another region for processing, and expand possible processing routes.

### 4.2 The Barium Lanthanide Copper Oxide Systems

#### 4.2.1 BaO-R_2_O_3_-CuO Prepared Under Air

Since the mid-eighties, an effort to understand the effect of lanthanide substitution on the properties and processing parameters of the high *T*_c_ superconductors BaR_2_Cu_3_O_6+_*_x_*. The progressive reduction in size of the lanthanide, which is known as the “lanthanide contraction”, allows us to systematically study the trend of crystal chemistry, solid solution formation, and phase equilibria in the system BaO-R_2_O_3_-CuO*_x_* as a function of the size of lanthanide ion, R^3+^.

**Fig. 30:**
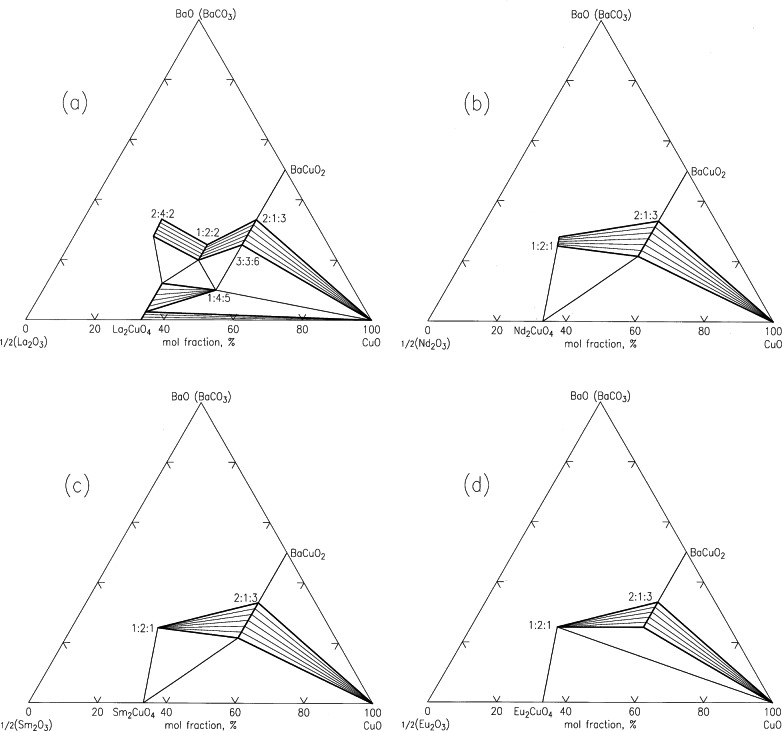

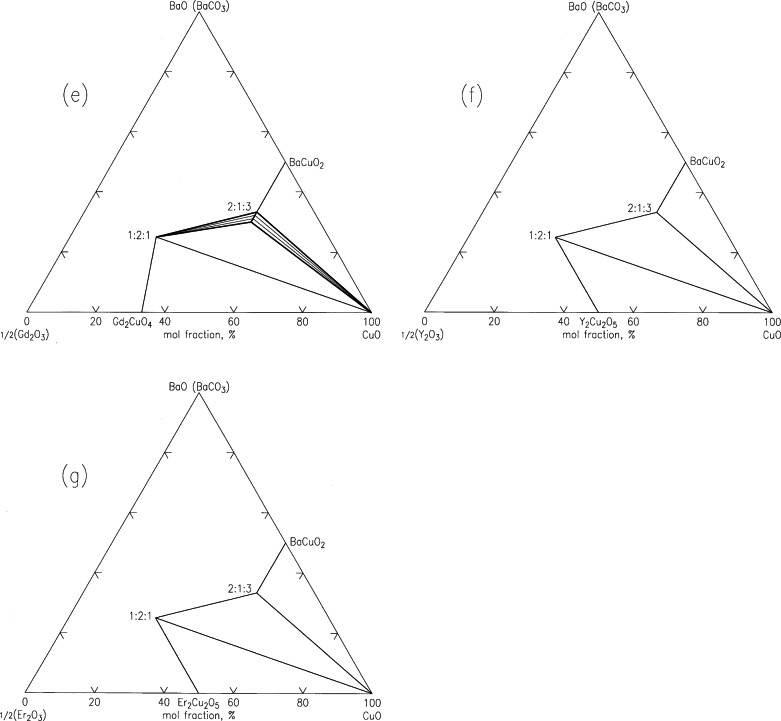
Subsolidus phase compatibility diagrams of BaO-½R_2_O_3_-CuO*_x_* near the CuO-rich region at 950 °C in air for (a) La, (b) Nd, (c) Sm, (d) Eu, [101]. Subsolidus phase compatibility diagrams of BaO-½R_2_O_3_-CuO*_x_* near the CuO-rich region at 950 °C in air for (e) Gd, (f) Y, and (g) Er [101].

The ternary phase compatibility diagrams of the systems BaO(BaCO_3_)-½Y_2_O_3_-CuO and BaO(BaCO_3_)-½R_2_O_3_-CuO systems in the vicinity of the CuO corners, where R = La, Nd, Sm, Eu, Gd, Er are shown schematically in Fig. 30 [101]. Several features of the progressive changes in the appearance of these ternary diagrams near the CuO corner are: (1) the La system has the largest number of ternary compounds and solid-solution series; this number decreases as the size of R decreases. (2) the superconductor phase, Ba_2_RCu_3_O_6+_*_x_*, for the first half of the lanthanide family, i.e., R = La, Nd, Sm, Eu and Gd, which are relatively larger in size, exhibit a solid solution of Ba_2−_*_z_*R_1+_*_z_*Cu_3_O_6+_*_x_* with a range of formation which decreases as the size of R decreases. The size compatibility between Ba^2+^ and R^3+^ is a predominant factor governing the formation of this solid solution. As the mismatch between R^3+^ and Ba^2+^ increases, the range of substitution decreases. (3) a trend is observed regarding the tie-line connections between BaR_2_CuO_5_, CuO, the superconductor phases Ba_2−_*_z_*R_1+_*_z_*Cu_3_O_6+_*_x_*, and the binary phase R_2_CuO_4_, or R_2_Cu_2_O_5_; note on Fig. 30(f) that the binary phase R_2_CuO_4_ is replaced by the binary phase R_2_Cu_2_O_5_ after the tie-line connection changes.

#### 4.2.2 BaO-Nd_2_O_3_-CuO Prepared Under CO_2_-Free Conditions

Subsolidus phase equilibria of the BaO-Nd_2_O_3_-CuO*_x_* system at *p*_O2_ = 100 Pa (0.1 % O_2_) and at *p*_O2_ = 21 kPa (21 % O_2_) were investigated by applying controlled atmosphere methods to minimize the presence of carbonate and CO_2_ and H_2_O contamination [102]. Under carbonate-free conditions, these two diagrams are similar to one another (except for differences in the extent of the solid solutions), but substantially different from those prepared in air. The system consists of three solid solutions and one stoichiometric ternary compound. At *p*_O2_ = 21 kPa, a compositionally dependent phase change was detected in the high *T*_c_ series, Ba_2−_*_x_*Nd_1+_*_x_*Cu_3_O*_z_*, from tetragonal (0.7 > *x* ≥ 0) to orthorhombic (≈1.0 ≥ *x* ≥ 0.7). The “brown-phase” Ba_1+_*_x_*Nd_2−_*_x_*CuO*_z_*, has a narrow homogeneity region. In the high BaO part of the phase diagram, a solid solution (Ba_2−_*_x_*Nd*_x_*)CuO_3+_*_z_* was confirmed. Ba_4_Nd_2_Cu_2_O*_z_* is an insulator, with a structure comprised of unusual CuO_5_ linear chains. Under carbonate-free conditions, a Ba_2−_*_x_*Nd_1+_*_x_*Cu_3_O_6+_*_z_ −* (Ba,Nd)_2_CuO_3−_*_x_* tie line was established, which substantially expands the field of stability of the Ba_2−_*_x_*Nd_1+_*_x_*Cu_3_O_6+_*_z_* superconductor phase into the BaO-rich region of the phase diagram.

#### 4.2.3 Orthorhombic to Tetragonal Phase Transformation of Ba_2_RCu_3_O_6+_*_x_*

It is well known that the 213-type phase undergoes phase transition as a function of oxygen content. Oxygen stoichiometry is important in determining the properties of superconductors. The phase transformation between the orthorhombic and tetragonal structures of six high *T*_c_ superconductors, Ba_2_RCu_3_O_6+_*_x_*, where R = Sm, Gd, Y, Ho, Er and Nd, and *x* = 0 to 1, have been determined [103,104]. The transformation from the oxygen-rich orthorhombic phase to the oxygen-deficient tetragonal phase involves two orthorhombic phases. The structural phase transition temperatures, oxygen stoichiometry and characteristics of the *T*_c_ plateaus appear to follow a trend anticipated from the dependence of the ionic radius on the number of *f* electrons as R progresses across the lanthanide series. Lanthanide elements with a smaller ionic radius stabilize the orthorhombic phase to higher temperatures and lower oxygen content. Also, the superconducting temperature is less sensitive to the oxygen content for materials with smaller ionic radius. A superlattice cell caused by oxygen ordering, with *a*′ = 2*a*, was observed for materials with smaller ionic radius.

### 4.3 Sr-R-Cu-O Systems

Since Ba and Sr are both alkaline-earth elements, studies of phase equilibria of the Sr-substituted systems may provide further understanding of the crystal chemistry of these lanthanide cuprates. While the Sr-213 phase Sr_2_RCu_3_O_6+_*_x_* is not stable in the Sr-system under ambient conditions, it can be stabilized either under high pressure [105], or when Cu at the basal chain site is partially or completely replaced by appropriate metals [106]. The phase formation, crystallography and crystal chemistry of the Sr_2_R(Cu,M)_3_O_6+_*_x_* (2112) structure (M = Al, Ga, Ta, and Nb, and R = La, Pr, Nd, Sm, Eu, Gd, Dy, Ho, Er, Tm, Yb, and Lu) have been systematically studied at NIST [107,108].

**Fig. 31:**
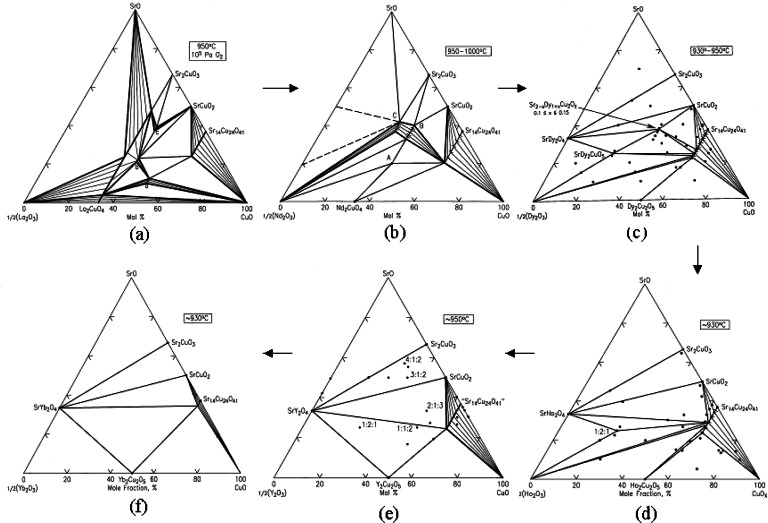
Phase diagrams of the SrO-R_2_O_3_-CuO systems, (a) R = La, (b) R = Nd, (c) R = Dy, (d) R = Ho, (e) R = Y, and (f) R = Yb [108, and references cited].

The phase diagrams of the SrO-R_2_O_3_-CuO*_x_* systems [108], where R = La, Nd, Dy, Y, Ho and Yb are shown in Fig. 31. A trend similar to that identified in the Ba-containing systems can be observed: the number of phases formed correlates with the size of R-ions. Larger lanthanide ions result in more complicated phase diagrams with a greater number of ternary phases.

**Fig. 32:**
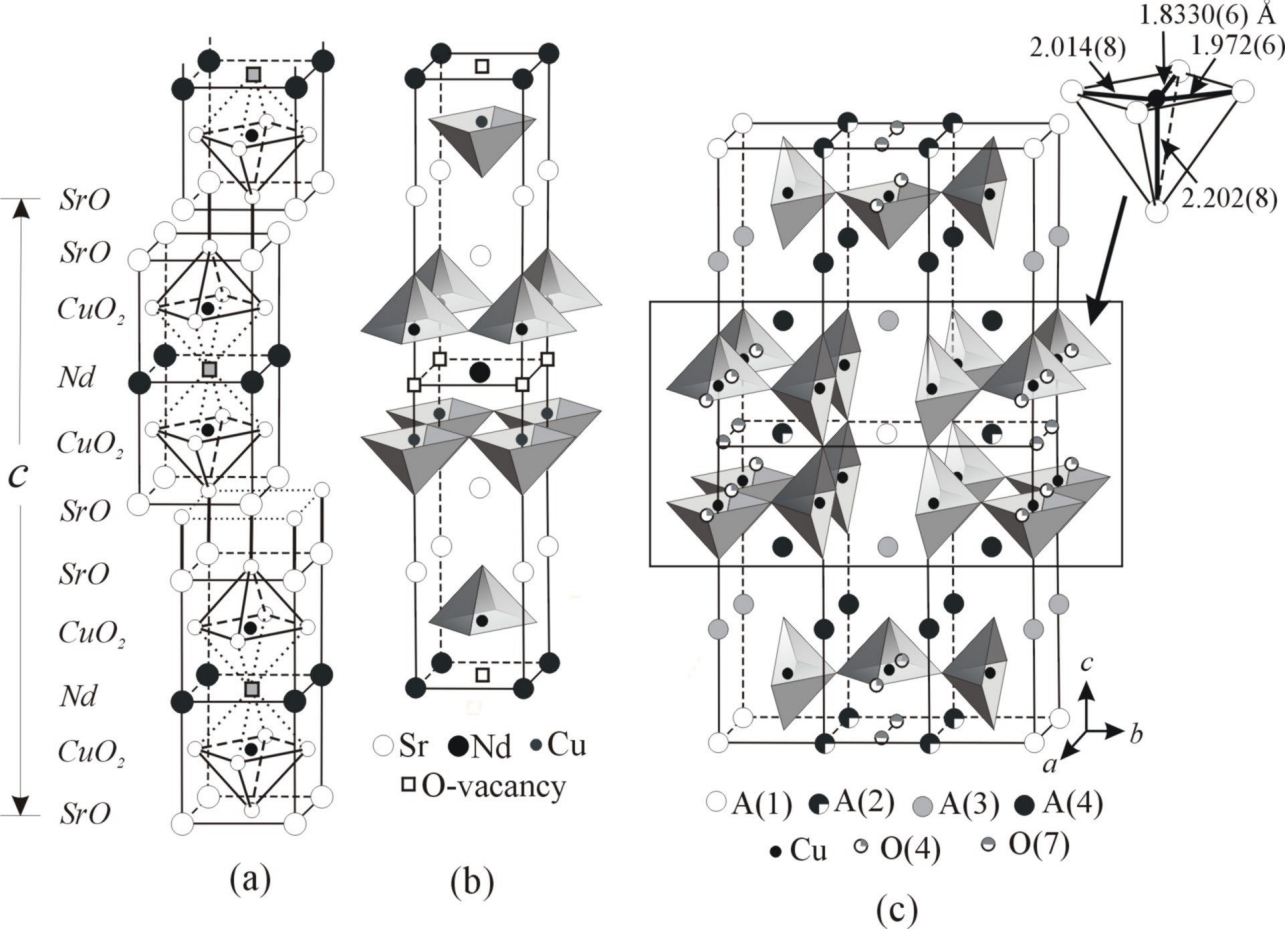
Structure of (a) Sr_3_Ti_2_O_7_ (b) Sr_1+_*_x_*R_2−_*_x_*Cu_2_O*_z_* (122) and Sr_2−_*_x_*R_1+_*_x_*Cu_2_O*_x_* (212) phase [108]. Cations in sites 1 and 3 are mainly occupied by the lanthanides, while site 2 is predominantly occupied by Sr.

In these systems, the structure of two related phases, Sr_2−_*_x_*R_1+_*_x_*Cu_2_O*_z_* (212) and Sr_1+_*_x_*R_2−_*_x_*Cu_2_O*_z_* (122) (R = Nd and Dy), are of particular interest because of the presence of layers of double-copper oxide pyramids [Fig. 32(b) and 32(c)]. The cation positions in these compounds are the same, only the oxygen distributions are different, and the lattice parameter *b* was found to be *b*_212_ ≈ 3*b*_122_. The structure of the tetragonal 122 phase (*I*4/*mmm*, *a* = 3.838401 Å and *c* = 19.647692 Å for SrNd_2_Cu_2_O*_z_*) is derived from Sr_3_Ti_2_O_7_ [Fig. 32(a)], which consists of a single rock salt layer alternating with two perovskite layers. Oxygen vacancies are found in the 122 perovskite unit, leading to square-pyramid copper coordinations and extended CuO planes. The structure of the orthorhombic (*Immm*) Nd- and Dy-212 analog is shown in Fig. 32(c). The perovskite slab [highlighted in Fig. 32(c)] features a structure motif of the 213 superconductor. While in 213 all Cu-O layers are infinite, the Cu-O units in the 212 structure are continuous only in the *b*-direction.

### 4.4 Bi-Pb-Sr-Ca-Cu-O System

Superconducting bismuth cuprates, which exhibit variations of cation ratio, form a family of layered-structure phases with ideal formulas Bi_2_Sr_2_Ca*_n_*_−1_Cu*_n_*O_4+2_*_n_* (*n* = 1, 2, and 3) (BSCCO). Three well known superconductor phases in the BSCCO system are commonly referred to (in the order of Bi:Sr:Ca:Cu) as the 1-layered 20K 2201, the 2-layered 80K 2212, and the 3-layered 110K 2223 phase. Among them, the two most widely investigated ones are the 2212 [109] and the Pb-doped 2223 ((Bi,Pb):Sr:Ca:Cu) phase [110–111]. These superconductors showed promising superconducting and other properties which are appropriate for wire and tape applications.

Bismuth forms peculiar and complicated structures partly due to the presence of the inert lone pair electrons. Chemical bonding in complexes with inert pair *s*^2^ ions is largely determined by *s*–*p* mixing [112]. In Bi^3+^, 6*s*–6*p* hybridization results in a pair of electrons being pushed off to one side of Bi so that the strong bonds are on the opposite side. These lone pair electrons affect the stereochemistry and become one of the common features of the Bi-containing compounds in the BSCCO system. In many compounds these lone pairs occupy channels in the structure. Bi is in general found to coordinate to from three to six oxygen atoms.

#### 4.4.1 Subsolidus Ternary Diagrams

Roth and co-workers completed the various binary and ternary diagrams: SrO-CuO, SrO-Bi_2_O_3_, SrO-Bi_2_O_3_-CuO, CaO-Bi_2_O_3_-CuO, SrO-CaO-Bi_2_O_3_, SrO-Bi_2_O_3_-CuO, CaO-CuO, CaO-Bi_2_O_3_, CaO-Bi_2_O_3_-CuO, SrO-CaO-Bi_2_O_3_. Examples of new phases whose structures have been determined are Ca_1−_*_x_*CuO_2_, Sr_14_Cu_24_O_41_, (Sr_0.16_Ca_0.84_)CuO_2_, Ca_4_Bi_6_O_13_, Sr_2_Bi_2_O_5_, Ca_6_Bi_6_O_15_, CaBi_2_O_4_, Sr_2_Bi_2_CuO_6_, SrBi_2_O_4_, Bi_14_(Sr, Ca)_12_O*_x_*, and Bi_2_(Sr, Ca)_4_O*_x_*.

##### 4.4.1.1 SrO-CaO-CuO

**Fig. 33:**
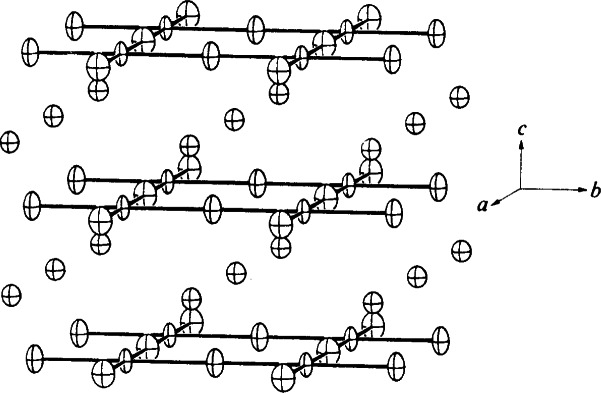
Extended view of (Ca_0.86_Sr_0.14_)CuO_2_. Only the Cu-O bonds are drawn [114].

In this system, mole fraction compositions with less than 33.3 % CuO were not investigated [113]. Three solid solutions were determined: Sr_2−_*_x_*Ca*_x_*CuO_3_, Sr_1−_*_x_*Ca*_x_*CuO_2_ and Sr_14−_*_x_*Ca*_x_*Cu_24_O_41_. A new ternary phase (Sr*_x_*Ca_1−_*_x_*)CuO_2_ (*x* ≈ 0.15) was determined [114]. This phase has an interesting structure (Fig. 33) because it can be regarded as the parent structure of A_2_B_2_Ca*_n_*_−1_Cu*_n_*O_4+2_*_n_* for very large *n*, where A = Bi, Tl; B = Ba, Sr. Ca_0.86_Sr_0.14_)CuO_2_ was found to be tetragonal with space group *P*4/*mmm*, and *a* = 3.8611(2) Å, and *c* = 3.1995(2) Å. This simple perovskite with a regular coordination for Ca, Sr and Cu is built from square-planar CuO_2_ sheets that sandwich Ca and Sr ions. The high symmetry and small unit cell can be considered as a result of deleting the bismuth oxide layers from the superconductor structure.

##### 4.4.1.2 SrO-Bi_2_O_3_-CuO

**Fig. 34:**
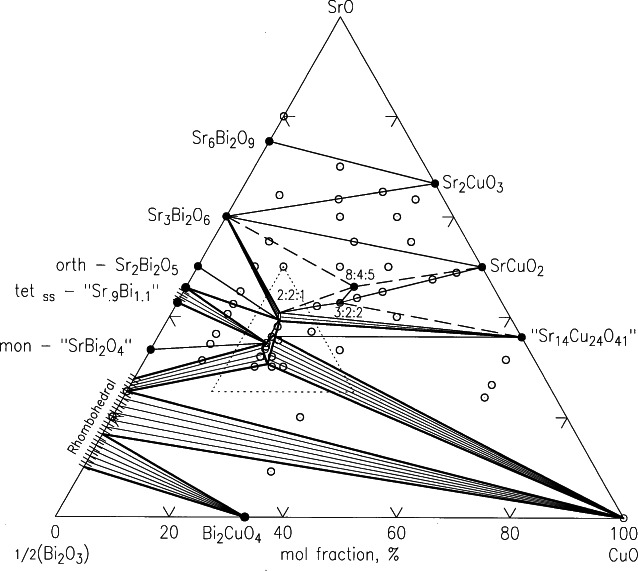
Phase diagram for the system SrO-½Bi_2_O_3_-CuO. (o)-compositions studied, (•)-compounds. This diagram represents subsolidus conditions, although Bi_2_O_3_ melts at 825 °C and therefore partial melting occurs below 875 °C in most compositions below the join CuO-Rhombohedral Solid Solution [115].

Four ternary oxide compounds were found (Fig. 34) [115]. Note the co-existence of the solid solution Bi_2.2−_*_x_*Sr_1.8+_*_x_*CuO*_z_* (commonly referred to as the Raveau 11905 phase) and the Bi_2_Sr_2_CuO_6_ (2201) phase. It is this 11905 phase that is the 1-layered 9K to 20K superconductor. The Raveau phase and the 2201 phase are in equilibrium with each other. The Raveau solid solution was found to extend from approximately 0.0 < *x* ≤ 0.15 for Sr_1.8−_*_x_*Bi_2.2+_*_x_*Cu_1−_*_x_*_/2_O*_z_*, and is structurally similar to the *n* = 1 member of Sr_2_Bi_2_Ca*_n_*_−1_Cu*_n_*O_2_*_n_*_+4_. Throughout the literature, the 2201 symbol is commonly used in place of the Raveau phase, and may be interpreted as a part of the extended single phase region of the Raveau phase.

**Fig. 35:**
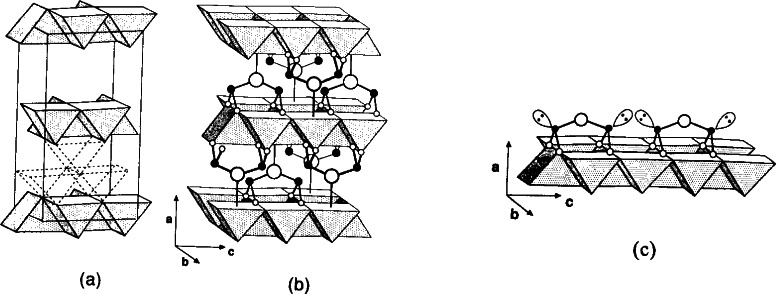
(a) The unit cell of Sr_2_Bi_2_O_5_ showing the SrO_6_ trigonal prisms of Sr_2_O_4_ (dotted polyhedra would complete the NiAs-type structure), and (b), position of [Sr_2_O_4_^4+^] groups, and (c), schematic diagram showing the lone-pair electrons [116].

In this system, several compounds were found to adopt unusual structure. For example, in the structure of Sr_2_Bi_2_O_5_ [116], all Bi atoms are in unusual threefold coordination with respect to oxygen. The compound crystallizes with the space group *Amam*, and with cell parameters of *a* = 6.1715(6) Å, *b* = 14.3066(13) Å, *c* = 3.8261(4) Å, and *Z* = 2 (PDF 39-1472 [1]). The basic structure consists of the intergrowth of two simple structural elements. The first element is the anti-nickel arsenide structure as shown in Figure 35(a). Each Sr is surrounded by six nearest neighbors in a trigonal prismatic arrangement. The second structural element can be regarded as Bi_2_O^4+^ chains which are inserted between the anti-NiAs slabs, as shown in Figure 35(b). Bi in 3-coordination with oxygen appears to exist only in systems containing the electropositive alkaline-earth cations Ca and Sr. These Sr and Ca atoms appear to allow the Bi-O bonds to be more covalent in nature and therefore more directional. Ordering of oxygen and vacancies is also visible along the *c*-direction and results in displacements of the Bi atoms along *c*. The lone pair electrons associated with the Bi can be envisioned as being directed toward the vacant site as schematically shown in Figure 35(c).

##### 4.4.1.3 SrO-CaO-Bi_2_O_3_

**Fig. 36:**
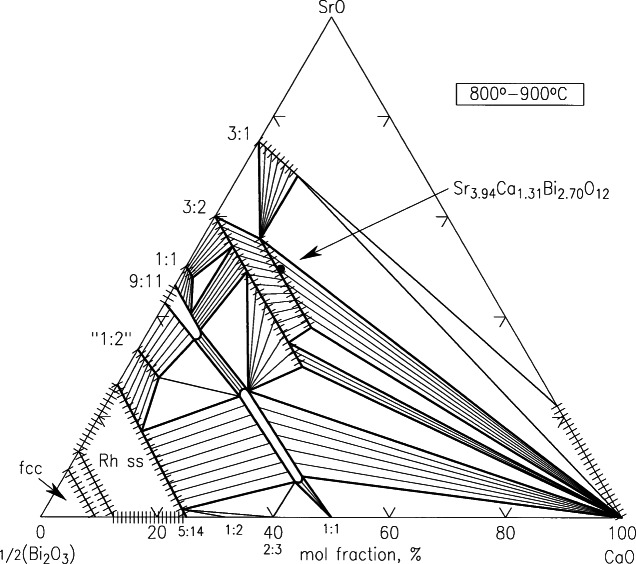
Phase diagram of the system ½Bi_2_O_3_-SrO-CaO (with experimental data points) showing the solid solution region for Bi_16_(Sr,Ca)_14_O_38_ and Bi_2_[Sr,Ca]_4_O*_x_* [117].

A large number of solid solution series appear in this system at 800 °C to 900 °C due to the substitution of the smaller Ca^+2^ for the larger Sr^+2^, as shown in Fig. 36 [117]. The phases of composition SrO: ½Bi_2_O_3_ of 3:1, 3:2, 1:1, 9:10, 1:2 accept CaO to form solid solutions to different extents. The rhombodehral solid solution (Rh_ss_) is complete across the entire range of SrO:CaO ratios. No SrO has been found in solid solution in either Ca_4_Bi_6_O_13_ or Ca_2_Bi_2_O_5_. Two ternary new phases (solid solutions) were discovered. The one with higher Sr content can be described with a general formula A_4_Bi_2_O*_x_*, and the other one is of general formula A_2_Bi_2_O*_x_*.

**Fig. 37:**
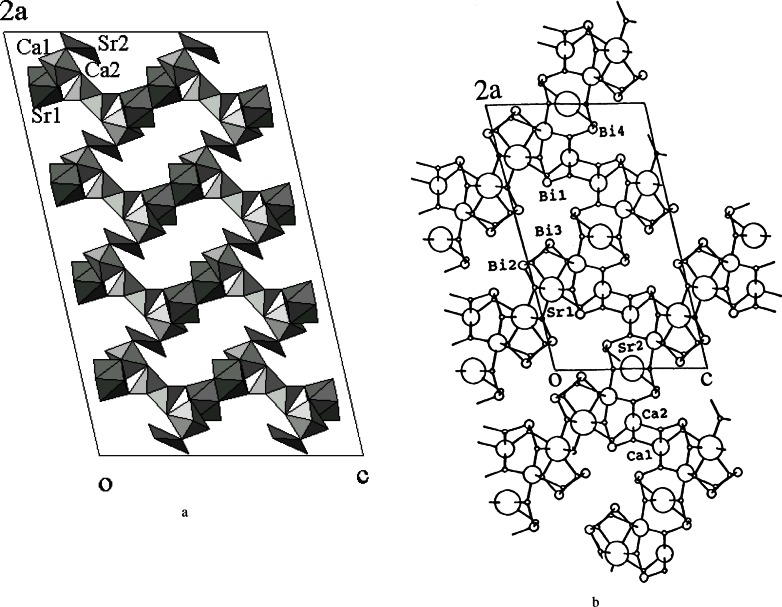
(a) Three-dimensional alkaline-earth oxide polyhedron network of Bi_16_Sr_5.44_Ca_8.56_O_38_ showing broad channels, and (b) network of Bi_16_Sr_5.44_Ca_8.56_O_38_ showing concentration of Bi’s in the channels [119].

A_2_Bi_2_O*_x_* (Bi_16_(Sr,Ca)_14_O_38_) crystallizes in the mono-clinic space group *C*2/*m*. A particular composition, Bi_16_Sr_5.44_Ca_8.56_O_38_, was found to have the cell parameters *a* = 21.764(4) Å, *b* = 4.3850(13) Å, *c* = 12.905(3) Å, *β* = 102.7(2) [118,119]. The Bi positions can be considered as the apices of highly distorted pyramids situated in the channels formed by a network of mixed alkaline-earth oxide polyhedra [Fig. 37(a) and 37(b)]. Two out of four crystallographically distinct Bi ions form infinite Bi-O zigzag ribbons of edge-linked Bi-O units, while each of the other two form oxygen corner-shared Bi-O chains along *b*. This structure exhibits similar features to Sr_2_Bi_2_O_5_ in that they both consist of stacked layers of metal atoms. Tunnels were found between layers which are occupied by Bi lone-pair electrons.

**Fig. 38:**
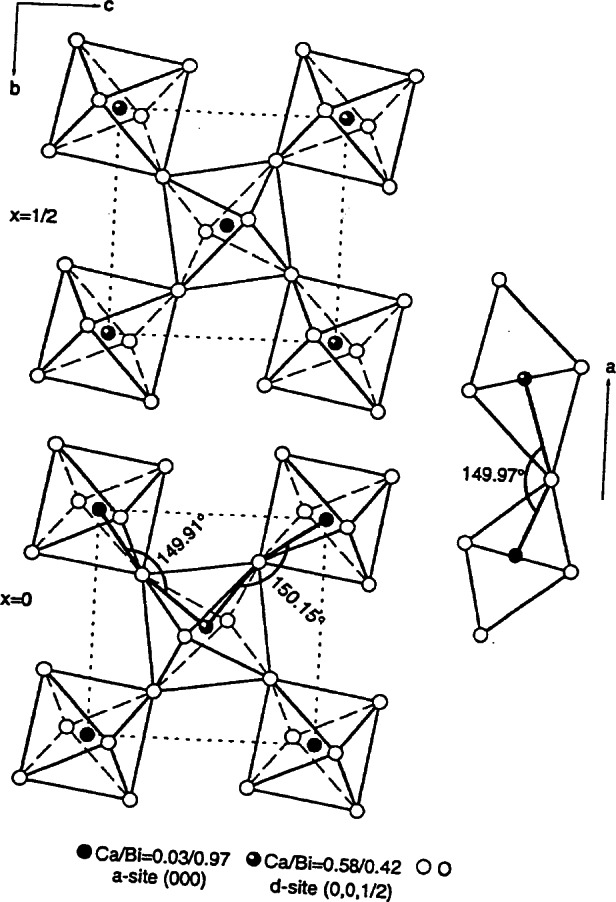
Schematic drawing of the structure of LT-Sr_3.94_Ca_1.31_Bi_2.70_O_12_ showing the M-O-M and M′-O′M′ angles of ≈ 15° about all three axis. The view is approximately along *b* [120].

Structural information of Bi_2_(Sr,Ca)_4_O*_x_* is important for BSCCO processing because this phase is in equilibrium with the 2201, 2212, and 2223 superconductors. The Bi_2_(Sr,Ca)_4_O*_x_* solid solution exists in both low-temperature (LT) oxidized form and high-temperature (HT) form. The structure of the LT- form (black) is monoclinic (pseudo-orthorhombic (Fig. 38), with space group *P*2_1_/*n* [120]. Structural refinements using neutron data gives the lattice parameters of (Bi_34_Sr_49.5_Ca_16.5_O_151_) as: *a* = 8.38931(16) Å, *b* = 5.99445(11) Å, *c* = 5.89572(11) Å, *β* = 89.961(3)°, and *V* = 296.49(1) Å^3^. This distorted perovskite (described in the perovskite ABO_3_ formula as Sr(Bi_0.7_Ca_0.3_)O_3_) features the 1:1 ordering of the M-site cations, resulting in the formula A_2_MM′O_6_. The MO_6_ and M′O_6_ octahedra are connected to each other via corner-sharing and are tilted with respect to the neighboring layers with an angle of ≈ 15° around all three axis. The tilt system symbol is *a*^+^*a*^−^*a*^−^ according to Glazer notation. All Bi ions are in the +5 oxidation state. In this 1:1 ordered structure, the A sites are solely occupied by Sr, the M-sites are mainly by Bi (2 % Ca), while on the *M*′ sites Bi and Ca are distributed in an approximate ratio of 3:2.

#### 4.4.2 Primary Crystallization Field

The presence of melt has been reported to improve the grain alignment and associated properties of high *T*_c_ superconductors in the (Bi,Pb)-Sr-Ca-Cu-O (BSCCO) system [121–122]. In this regard, the powder-in-tube (PIT) processing technique, which involves melt processing, has been found to be a feasible technique for the large-scale production of wires and tapes [123]. Because of the fact that the high *T*_c_ phases in the BSCCO systems, similar to the Ba-R-Cu-O systems, do not melt congruently, for crystal growth or grain growth, it is essential to obtain primary phase information. In the mid-nineties, Wong-Ng and Cook, in collaboration with Kearsley, successfully determined the phase relationship and the primary phase fields of the Pb-free 2212 phase, the (Bi,Pb)-2223 phase, and effect of Ag on (Bi,Pb)-2223.

##### 4.4.2.1 2212 Superconductor

**Fig. 39:**
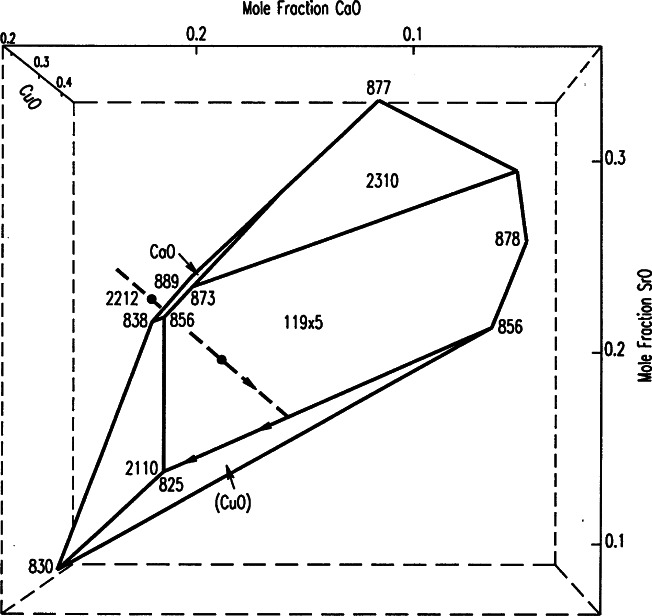
Primary crystallization field of the 2212 phase in the BSCCO system. A proposed crystallization path is also shown [109].

A total of 10 phases was found to be in equilibrium with the 2212 phase [109]. For convenience, symbols (in the order Bi:Sr:Ca:Cu) are used to represent the ternary and quaternary oxide phases. The equilibrium phases were 0×21 [(Ca,Sr)_2_CuO_3_], 11 9 × 5 [(Bi,Pb)_2.2_Sr_1.8−_*_x_*Ca*_x_*CuO*_z_*], 2110 [Bi_16_(Sr,Ca)_14_O*_z_*], 0 14 × 24 [(Sr,Ca)_14_Cu_24_O_41_], 2310 [Bi_2_(Sr,Ca)_4_O*_z_*], 4805 [Bi_4_Sr_8_Cu_5_O*_z_*), 2201 [(Bi,Pb)_2_Sr_2−_*_x_*Ca*_x_*CuO*_z_*], (Ca,Sr)O, CuO, and 0×11 [(Sr_1−_*_x_*Ca*_x_*)CuO_2_, Ca-rich]. Sixteen self-consistent 4-phase equilibrium volumes containing 2212 as a member were determined. The minimum melt compositions of each of these volumes were used to construct the primary phase field (Fig. 39). In this diagram the volume is expressed in Cartesian coordinates and the cotectic faces that are visible in the orientation shown are labeled. The initial melting temperatures are indicated at the corners of the volume. Inside the volume, the 2212 phase is in equilibrium with liquid (L). On each face 2212 is in equilibrium with L and the labeled phase. Along the edges, 2212 is in equilibrium with two phases plus L, and at the corners, 2212 is in equilibrium with three phases with L. For crystal growth, one should use the composition inside the volume. The crystallization path for a selected composition (Bi:Sr:Ca:Cu = 26.9: 24.7: 17.6:30.8) is shown. During crystal growth, temperature continues to drop, and when the path is inside the volume, only 2212 crystal crystallizes out. When the path intersects the face and the edge, the 11 9 × 5 phase and CuO also crystallize, and eventually at the 2212 eutectic temperature, 2110 also appears. Large grains of 2212 can be obtained using this composition. However, if the composition is outside this field even slightly, large second-phase impurities would interfere with processing and have a detrimental effect on properties [124].

##### 4.4.2.2 (Bi,Pb)2223

**Fig. 40:**
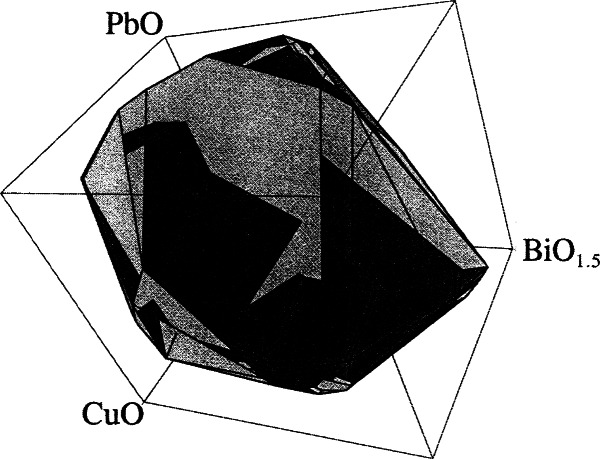
The primary crystallization field of the Pb-2223 phase. An isoplethal section made by holding the SrO and CaO values constant at the medium mole fraction percent for the 29 data points (SrO = 20.4, and CaO = 6.9) [110].

**Fig. 41:**
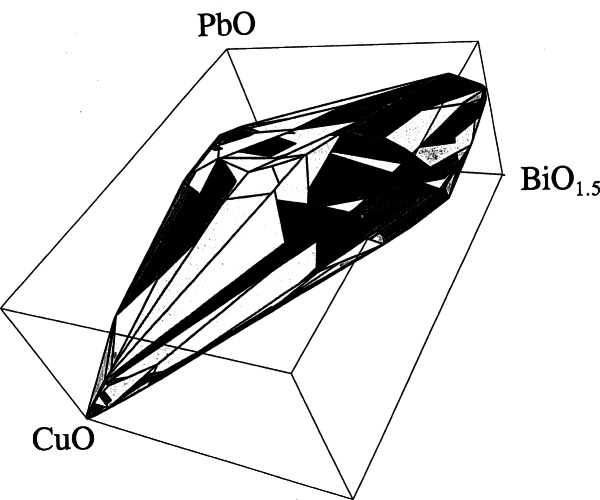
An isoplethal section of the primary crystallization field of the Pb-2223 phase made by holding the SrO, CaO, and Ag values constant at the medium values for the 29 data points (mole fraction percent of SrO, CaO, and Ag = 22.8, 20.1, and 3.3, respectively) [111].

(Bi,Pb)-2223 was found to be in equilibrium with 11 phases, including (Ca,Sr)O, CuO, 0×21, 2201, 11 9 × 5, 1×20 ((Ca,Sr)_2_PbO_4_), 0 14 × 24, 2310, 0×11 (Ca-rich), 3221((Pb,Bi)_3_Sr_2_Ca_2_CuO*_x_*), 0×11′ (Ca-poor). In these symbols, × is used to represent the amount of mutual substitution of the Ca and Sr sites. At 810 °C to 820 °C in volume fraction of 7.5 % O_2_ (92.5 % Ar), 29 five-phase volumes that involve the (Bi,Pb)-2223 phase were found to be mutually stable in a topologically consistent manner [110]. The (Bi,Pb)-2223 primary phase field was constructed using the initial melts of these 29 five-phase volumes, and was modeled using the convex hull technique. It is described by the matrix equation: ***Ax*** − ***b*** ≤ 0, where ***A*** is a matrix whose rows define the unit normal vectors to the faces of the convex hull. Each element of the vector ***b*** defines the proximity of the given face to the origin. The vector ***x*** gives the coordinates corresponding to a given point. Figure 40 shows a projected section made by holding SrO and CaO constant at the median values of the data points. The presence of Ag basically does not alter the subsolidus relationships, but the shape of the primary phase field (Fig. 41) is much flattened along the PbO direction, indicating the Pb composition in liquid is reduced due to Ag [111]. The melting temperatures of the volumes due to the presence of Ag are consistently lower, from a range of 2 °C to 25 °C. Ag is present in liquids from 1 % to 6 % mole fraciton. The dissolution of Ag in melt implies that dissolution of Ag tubing may occur during melt processing.

### 4.5 Tl-Containing Systems

**Fig. 42:**
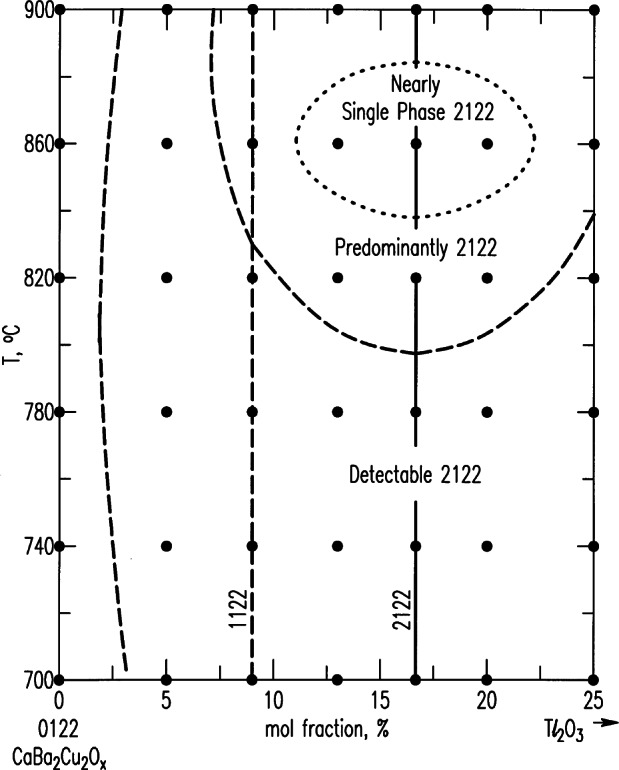
Temperature-composition plot showing experiments completed under an oxygen atmosphere, with extent of 2212 (Tl:Ca:Ba:Cu) phase formation [125].

Phase equilibrium studies in the Tl-containing system are not as extensive as those in the BaO-Y_2_O_3_-CuO, or the BSCCO systems, partly because of the additional processing parameters of vapor pressure, and also because of the toxicity of the Tl-containing compounds. At NIST, a smaller-scale program was conducted by L. P. Cook and co-workers in the mid-nineties. As a result of this work, the stability of the 2212 (Tl:Ca:Ba:Cu) phase (n = 2 member of the Ruddelsden-Popper homologous series, Tl_2_Ba_2_Ca_n−1_Cu_n_O_2n+3_) and the solid solution extent was obtained [125]. Figure 42 illustrates the solid solution extent of the 2212 phase in the Ba_2_CaCu_2_O_2_-Tl_2_O_3_ system. It was found that a sample with a maximum *T*_c_ occurred with less Tl_2_O_3_ than the 2212 stoichiometry.

The properties and stability of the TlSr_2_Ca_2_Cu_3_O_x_ (1223) family of phases can be improved by creating pinning centers by partial substitution of Pb and/or Bi for Tl in the rock salt layer, and substitution of Ba for Sr in the perovskite layer. The melting and vaporization of the 1223 phase have been investigated using a combination of dynamic methods (differential thermal analysis, thermogravimetry, and effusion) and post-quenching characterization techniques (scanning electron microscopy, energy dispersive x-ray spectrometry, and powder x-ray diffaction) [126]. The melting and vaporization equilibria of the 1223 phase are complex, with as many as seven phases participating simultaneously. The melting reaction was found to be nominally as: 1223 → 1212 + (Ca,Sr)_2_CuO_3_ + (Sr,Ca)CuO_2_ + (Sr,Ca)CuO_2_ + BaPbO_3_ + (Ca,Sr)O + L.

### 4.6 High Pressure Studies of High *T*_c_ Superconductors

Mechanical reliability is critical for the successful applications of high *T*_c_ materials. To estimate toughness of a material quantitatively, it is necessary to know the value of Young’s modulus for the solid, which can in turn be obtained from the bulk modulus, or its inverse, the compressibility, obtained from high pressure x-ray powder diffraction experiments [127–129]. Both the high *T*_c_ superconductor Ba_2_YCu_3_O_7_ and the orthorhombic “green phase” BaLu_2_CuO_5_ feature anisotropic compression. The Ba_2_YCu_3_O_7_ structure has perovskite-like CuO layers and chains running perpendicular to the *c*-direction [130]. The largest compression occurred perpendicular to these CuO layers (2.3 %). The least compression was found to occur within the perovskite-like layers because of the oxygen packing [(2 % in the *a*-direction (oxygen absent in the basal plane) and 1.1 % in *b*-direction (Cu-O chain direction)]. The framework of the green phase can be considered as built up from distorted monocapped trigonal prisms, RO_7_ blocks. Consecutive layers of the R prisms are stacked with shared edges to from wave-like chains parallel to the long *b*-axis [128]. The compressibility behavior of Ba_2_YCu_3_O_7_ is similar to BaLu_2_CuO_5_ in which greater compression occurs perpendicular to the layer direction (1.2 % in both *a*- and *b*-directions, and 1.5 % in the *c*-direction). As the bulk modulus of the green phase (BaLu_2_CuO_5_, 251 GPa) is significantly greater than that of Ba_2_YCu_3_O_7_ (reported values of bulk modulus range from 55 GPa to 196 GPa), there is a possibility that mixing the high *T*_c_ phase with the green phase (i.e., in fiber form) may result in improved toughness value of the material.

## 5. Collaboration With the American Ceramic Society—Compilation of Phase Diagrams

For over 60 years, there has been a long-standing cooperative effort between NBS/NIST and the American Ceramic Society (ACerS) to publish compilations of published phase diagrams [131]. This collaborative effort was initiated as early as in 1933 by H. Insley and F. P. Hall, and the diagrams were published by the ACerS. To date, more than 20 000 phase diagrams have been published. There are a total of thirteen volumes of compilations of oxides, salts, semiconductor elements, borides, carbides, and nitrides for structural ceramics; three annual volumes of diagrams published in 1991, 1992, and 1993. In addition, two volumes containing high-temperature superconductor data including diagrams, write-ups and bibliographic information were also published in 1991 and 1997 (Superconductors Volumes I and II). An additional volume of phase diagrams for zirconium and zirconia system was also published in 1998. The sources of these phase diagrams are mostly from the literature, and the data have been reviewed and edited by experts in the field. Under this collaborative effort, technical expertise in the compilation of phase diagrams is provided by NIST researchers, and by various university, industry, and government collaborators. The ACerS assumes the responsibility for the production aspects of the work and for all means of data dissemination.

These diagrams have been used extensively and over 45 000 copies of individual volumes have been sold. At the time of the ACerS centennial celebration in 1998, the publication of phase diagrams was cited as one of the two most important accomplishments of the Society affecting the development of ceramics. Today the project is under the leadership of T. A. Vanderah. In addition to the general editors (Robert S. Roth, H. McMurdie, Helen M. Ondik, and L. P. Cook), other individuals who also serve in various capacity to oversee the production of each volume include M. A. Harne, P. Schenck, K. Hill, C. Cedeo, M. Swanson, E. Farabaugh, and C. G. Messina.
